# 16S rRNA Amplicon Sequencing for Epidemiological Surveys of Bacteria in Wildlife

**DOI:** 10.1128/mSystems.00032-16

**Published:** 2016-07-19

**Authors:** Maxime Galan, Maria Razzauti, Emilie Bard, Maria Bernard, Carine Brouat, Nathalie Charbonnel, Alexandre Dehne-Garcia, Anne Loiseau, Caroline Tatard, Lucie Tamisier, Muriel Vayssier-Taussat, Helene Vignes, Jean-François Cosson

**Affiliations:** a INRA, CBGP, Montferrier sur Lez, France; b INRA, EpiA, Clermont-Ferrand, France; c INRA, Sigenae, France; d INRA, GABI, AgroParisTech, Université Paris-Saclay, Jouy-en-Josas, France; e IRD, CBGP, Montferrier sur Lez, France; f INRA, Bipar, Maisons-Alfort, France; g CIRAD, AGAP, Montpellier, France; New York University

**Keywords:** bacteria, emerging infectious diseases, high-throughput sequencing, metabarcoding, molecular epidemiology, next-generation sequencing, rodents, West Africa, zoonoses

## Abstract

The human impact on natural habitats is increasing the complexity of human-wildlife interactions and leading to the emergence of infectious diseases worldwide. Highly successful synanthropic wildlife species, such as rodents, will undoubtedly play an increasingly important role in transmitting zoonotic diseases. We investigated the potential for recent developments in 16S rRNA amplicon sequencing to facilitate the multiplexing of the large numbers of samples needed to improve our understanding of the risk of zoonotic disease transmission posed by urban rodents in West Africa. In addition to listing pathogenic bacteria in wild populations, as in other high-throughput sequencing (HTS) studies, our approach can estimate essential parameters for studies of zoonotic risk, such as prevalence and patterns of coinfection within individual hosts. However, the estimation of these parameters requires cleaning of the raw data to mitigate the biases generated by HTS methods. We present here an extensive review of these biases and of their consequences, and we propose a comprehensive trimming strategy for managing these biases. We demonstrated the application of this strategy using 711 commensal rodents, including 208 *Mus musculus domesticus*, 189 *Rattus rattus*, 93 *Mastomys natalensis*, and 221 *Mastomys erythroleucus*, collected from 24 villages in Senegal. Seven major genera of pathogenic bacteria were detected in their spleens: *Borrelia*, *Bartonella*, *Mycoplasma*, *Ehrlichia*, *Rickettsia*, *Streptobacillus*, and *Orientia*. *Mycoplasma*, *Ehrlichia*, *Rickettsia*, *Streptobacillus*, and *Orientia* have never before been detected in West African rodents. Bacterial prevalence ranged from 0% to 90% of individuals per site, depending on the bacterial taxon, rodent species, and site considered, and 26% of rodents displayed coinfection. The 16S rRNA amplicon sequencing strategy presented here has the advantage over other molecular surveillance tools of dealing with a large spectrum of bacterial pathogens without requiring assumptions about their presence in the samples. This approach is therefore particularly suitable to continuous pathogen surveillance in the context of disease-monitoring programs.

**IMPORTANCE** Several recent public health crises have shown that the surveillance of zoonotic agents in wildlife is important to prevent pandemic risks. High-throughput sequencing (HTS) technologies are potentially useful for this surveillance, but rigorous experimental processes are required for the use of these effective tools in such epidemiological contexts. In particular, HTS introduces biases into the raw data set that might lead to incorrect interpretations. We describe here a procedure for cleaning data before estimating reliable biological parameters, such as positivity, prevalence, and coinfection, using 16S rRNA amplicon sequencing on an Illumina MiSeq platform. This procedure, applied to 711 rodents collected in West Africa, detected several zoonotic bacterial species, including some at high prevalence, despite their never before having been reported for West Africa. In the future, this approach could be adapted for the monitoring of other microbes such as protists, fungi, and even viruses.

## Abstract (precis)

Several recent public health crises have shown that the surveillance of zoonotic agents in wildlife is important to prevent pandemic risks. High-throughput sequencing (HTS) technologies are potentially useful for this surveillance, but rigorous experimental processes are required for the use of these effective tools in such epidemiological contexts. In particular, HTS introduces biases into the raw data set that might lead to incorrect interpretations. We describe here a procedure for cleaning data before estimating reliable biological parameters, such as positivity, prevalence, and coinfection, using 16S rRNA amplicon sequencing on an Illumina MiSeq platform. This procedure, applied to 711 rodents collected in West Africa, detected several zoonotic bacterial species, including some at high prevalence, despite their never before having been reported for West Africa. In the future, this approach could be adapted for the monitoring of other microbes such as protists, fungi, and even viruses.

## INTRODUCTION

Pathogen monitoring in wildlife is a key method for preventing the emergence of infectious diseases in humans and domestic animals. More than half the pathogens causing disease in humans originate from animal species (1). The early identification of zoonotic agents in animal populations is therefore of considerable interest for human health. Wildlife species may also act as a reservoir for pathogens capable of infecting livestock, with significant economic consequences (2). The monitoring of emerging diseases in natural populations is also important for preserving biodiversity, because pathogens carried by invasive species may cause the decline of endemic species (3). There is, therefore, a need to develop screening tools for identifying a broad range of pathogens in samples consisting of large numbers of individual hosts or vectors.

High-throughput sequencing (HTS) approaches require no prior assumptions about the bacterial communities present in samples that are diverse in nature, including noncultivable bacteria. Such HTS microbial identification approaches are based on the sequencing of all (WGS [whole-genome sequencing]) or some (RNA-seq [whole-RNA sequencing] or 16S rRNA amplicon sequencing) of the bacterial DNA or RNA in a sample, followed by comparison to a reference sequence database (4). HTS has made major contributions to the generation of comprehensive inventories of the bacterial species, including pathogens, present in humans (5). Such approaches are now being extended to the characterization of bacteria in wildlife (6–13). However, improvements in the estimation of infection risks will require more than just the detection of bacterial pathogens. Indeed, we will also need to estimate the prevalence of these pathogens by host taxon and/or environmental features, together with coinfection rates (14, 15) and pathogen interactions (16, 17).

Razzauti et al. (8) recently used 16S rRNA amplicon sequencing with the dual-index sequencing strategy of Kozich et al. (18) to detect bacterial pathogens in very large numbers (up to several hundred samples in a single run) of rodent samples by the use of an Illumina MiSeq sequencing platform. The 16S rRNA amplicon sequencing technique is based on the amplification of small fragments of one or two hypervariable regions of the 16S rRNA gene. The sequences of these fragments are then obtained and compared with reference sequences in curated databases for taxonomic identification (4, 19). Multiplexed approaches of this kind include short indices (or tags) that are linked to the PCR products and specific to a given sample. This makes it possible to assign the sequences generated by the HTS run to a particular sample following bioinformatic analysis of the data set (18). Razzauti et al. (8) demonstrated the considerable potential of this approach for determining the prevalence of bacteria within populations and for analyzing bacterial interactions within hosts and vectors, based on the accurate characterization of bacterial diversity within each of the individual samples that it provides. However, various sources of error during the generation and processing of HTS data (20) may make it difficult to determine which samples are really positive or negative for a given bacterium. The detection of one or a few sequences assigned to a given taxon in a sample does not necessarily mean that the bacterium is actually present in that sample. We carried out an extensive literature review, from which we identified several potential sources of error involving all stages of a 16S rRNA amplicon sequencing experiment—from the collection of samples to the bioinformatic analysis—that might lead to false-negative or false-positive screening results (Table 1) (18, 19, 21–40). These error sources have now been documented, and recent initiatives have called for the promotion of open sharing of standard operating procedures (SOP) and best practices in microbiome research (21). However, no experimental designs minimizing the impact of these sources of error on HTS data interpretation have yet been reported.

**TABLE 1:** Sources of bias during the experimental and bioinformatic steps of 16S rRNA amplicon sequencing: consequences for data interpretation and solutions for mitigating these biases

Experimental step(s)	Source(s) of errors	Consequence(s)	Solution(s)
Sample collection	Cross-contamination between individuals (21)	False-positive samples	Rigorous processing (decontamination of the instruments, cleaning of the autopsy table, use of sterile bacterium-free consumables, gloves, masks)
			Negative controls during sampling (e.g., organs of healthy mice during dissection)
	Collection and storage conditions (21)	False-positive and false-negative samples	Use of appropriate storage conditions/buffers; use of unambiguously identified samples; double-checking of tube labeling during sample collection
			
DNA extraction	Cross-contamination between samples (22)	False-positive samples	Rigorous processing (separation of pre- and post-PCR steps, use of sterile hood and filter tips and sterile bacterium-free consumables)
	Reagent contamination with bacterial DNA (21, 23)	False-positive samples	Negative controls for extraction (extraction without sample)
	Small amounts of DNA (21, 24)	False-negative samples	Use of an appropriate DNA extraction protocol; discarding of samples with a low DNA concentration
			
Target DNA region and primer design	Target DNA region efficacy (19, 25)	False-negative samples due to poor taxonomic identification	Selection of an appropriate target region and design of effective primers for the desired taxonomic resolution
	Primer design (21, 26)	False-negative samples due to biases in PCR amplification for some taxa	Checking of the universality of the primers with reference sequences
			
Tag/index design and preparation	False assignments of sequences due to cross-contamination between tags/indices (27, 28)	False-positive samples	Rigorous processing (use of sterile hood and filter tips and sterile bacterium-free consumables, brief centrifugation before the opening of index storage tubes, separation of pre- and post-PCR steps)
			Negative controls for tags/indices (empty wells without PCR reagents for particular tags or index combinations)
			Positive controls for alien DNA, i.e., a bacterial strain highly unlikely to infect the samples studied (e.g., a host-specific bacterium unable to persist in the environment) to estimate false-assignment rate
	False assignments of sequences due to inappropriate tag/index design (29)	False-positive samples	Fixing of a minimum number of substitutions between tags or indices; each nucleotide position in the sets of tags or indices should display about 25% occupation by each base for Illumina sequencing
			
PCR amplification	Cross-contamination between PCRs (28)	False-positive samples	Rigorous processing (brief centrifugation before opening the index storage tubes, separation of pre- and post-PCR steps)
			Negative controls for PCR (PCR without template), with microtubes left open during sample processing
	Reagent contamination with bacterial DNA (21, 23)	False-positive samples	Rigorous processing (use of sterile hood and filter tips and sterile bacterium-free consumables)
			Negative controls for PCRs (PCR without template), with microtubes closed during sample processing
	Chimeric recombinations by jumping PCR (27, 30–33)	False-positive samples due to artifactual chimeric sequences	Increasing the elongation time and decreasing the number of cycles; use of a bioinformatic strategy to remove the chimeric sequences (e.g., Uchime program)
	Poor or biased amplification (44)	False-negative samples	Increasing the amount of template DNA; optimizing the PCR conditions (reagents and program)
			Use of technical replicates to validate sample positivity
			Positive controls for PCR (extraction from infected tissue and/or bacterial isolates)
Library preparation	Cross-contamination between PCRs/libraries (22)	False-positive samples	Rigorous processing (use of sterile hood and filter tips and sterile bacterium-free consumables, electrophoresis and gel excision with clean consumables, separation of pre- and post-PCR steps)
			Use of a protocol with an indexing step during target amplification
			Negative controls for indices (changing well positions between library preparation sessions)
	Chimeric recombinations by jumping PCR (27)	False-positive samples due to interindividual recombinations	Avoiding PCR library enrichment of pooled samples
			Positive controls for alien DNA, i.e., DNA from a bacterial strain that should not be identified in the sample (e.g., a host-specific bacterium unable to persist in the environment)
			
MiSeq sequencing (Illumina)	Sample sheet errors (21)	False-positive and negative samples	Negative controls (wells without PCR reagents for a particular index combination)
	Run-to-run carryover (Illumina technical support note no. 770-2013-046)	False-positive samples	Washing of the MiSeq with dilute sodium hypochlorite solution
	Poor quality of reads due to flow cell overloading (34)	False-negative samples due to low quality of sequences	qPCR quantification of the library before sequencing
	Poor quality of reads due to low-diversity libraries (Illumina technical support note no. 770-2013-013)		Decreasing cluster density; creation of artificial sequence diversity at the flow cell surface (e.g., by adding 5%–10% PhiX DNA control library)
	Small number of reads per sample (35, 36)	False-negative samples due to low depth of sequencing	Decreasing the level of multiplexing
			Discarding the sample with a low number of reads
	Too-short overlapping read pairs (18)	False-negative samples due to low quality of sequences	Increasing paired-end sequence length or decreasing the length of the target sequence
	Mixed clusters on the flow cell (27)	False-positive samples due to false index pairing	Use of a single barcode sequence for both the i5 and i7 indices for each sample (when possible, e.g., with a small number of samples)
			Positive controls for alien DNA, i.e., DNA from a bacterial strain highly unlikely to be found in the rodents studied (e.g., a host-specific bacterium unable to persist in the environment)
			
Bioinformatics and taxonomic classification	Poor quality of reads	False-negative samples due to poor taxonomic resolution	Removal of low-quality reads
	Errors during processing (sequence trimming, alignment) (18, 37, 38)	False-positive and false-negative samples	Use of standardized protocols and reproducible workflows
	Incomplete reference sequence databases (39)	False-negative samples	Selection of an appropriate database for the selected target region and testing of the database for bacteria of particular interest
	Error of taxonomic classification (40)	False-positive samples	Positive controls for PCRs (extraction from infected tissue and/or bacterial isolates and/or mock communities)
			Checking of taxonomic assignments by other methods (e.g., blast analyses using different databases)

**FIG 1:**
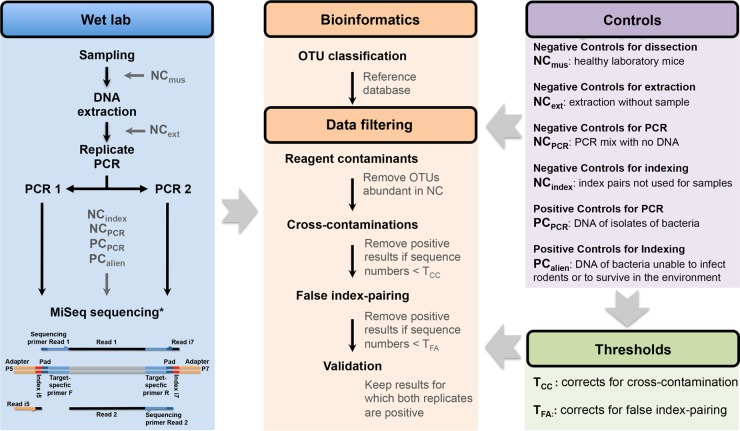
Workflow of the wet laboratory, bioinformatics, and data filtering procedures in the process of data filtering for 16S rRNA amplicon sequencing. Reagent contaminants were detected by analyzing the sequences in the NC_ext_ and NC_PCR_ controls. Sequence number thresholds for correcting for cross-contamination (T_CC_) are OTU and run dependent and were estimated by analyzing the sequences in the NC_mus_, NC_ext_, NC_PCR_, and PC_index_ controls. Sequence number thresholds for correcting for false-index-pairing (T_FA_) values are OTU and run dependent and were estimated by analyzing the sequences in the NC_index_ and PC_alien_ controls. A result was considered positive if the number of sequences was >T_CC_ and >T_FA_. Samples were considered positive if a positive result was obtained for both PCR replicates. *, see Kozich et al. (18) for details on the sequencing.

We describe here a rigorous experimental design for the direct estimation of biases from the data produced by 16S rRNA amplicon sequencing. We used these bias estimates to control and filter out potential false-positive and false-negative samples during screening for bacterial pathogens. We applied this strategy to 711 commensal rodents collected from 24 villages in Senegal, Western Africa: 208 *Mus musculus domesticus*, 189 *Rattus rattus*, 93 *Mastomys natalensis*, and 221 *Mastomys erythroleucus*. Pathogenic bacteria associated with the rodents were analyzed using a protocol based on Illumina MiSeq sequencing of the V4 hypervariable region of the 16S rRNA gene (18). We considered the common pitfalls listed in Table 1 during the various stages of the experiment (see details in the workflow procedure in Fig. 1). Biases in assessments of the presence or absence of bacteria in rodents were estimated directly from the data set by including and analyzing negative controls (NC) and positive controls (PC) at various stages of the experiment (see details in Materials and Methods) and by systematically using sample replicates. This strategy delivers realistic and reliable estimates of bacterial prevalence in wildlife populations and could be used to analyze the cooccurrence of different bacterial species within individuals.

## RESULTS AND DISCUSSION

### Raw sequencing results.

The sequencing of 1,569 PCR products in two MiSeq runs generated a total of 23,698,561 raw paired-end sequence reads (251 bp) of the V4 region of the 16S rRNA gene. Because we made PCR replicates for each rodent sample, and because we included several controls in each sequencing run, we had more PCR products (*N* = 1,569) than rodent samples (*N* = 711) (see summary in Table S1 in the supplemental material and complete information by sample and run in Table S2). Overall, 99% of the PCRs generated more than 3,000 raw reads (mean, 11,908 reads; standard deviation, 6,062 reads). The raw sequence files have been deposited in FASTQ format in the Dryad Digital Repository (http://dx.doi.org/10.5061/dryad.m3p7d) (41).

10.1128/mSystems.00032-16.5 Table S1  Numbers of samples and numbers of PCRs for wild rodents and controls. Negative controls for dissection, NC_mus_; negative controls for extraction, NC_ext_; negative controls for PCR, NC_PCR_; negative controls for indexing, NC_index_; positive controls for PCR, PC_PCR_; positive controls for indexing, PC_alien_. See also Fig. 1 and Materials and Methods for more details concerning negative controls (NC) and positive controls (PC). Download Table S1, PDF file, 0.1 MB. Copyright © 2016 Galan et al. 2016 Galan et al. This content is distributed under the terms of the Creative Commons Attribution 4.0 International license.

10.1128/mSystems.00032-16.6 Table S2  Information concerning samples and the composition of the sequencing runs. Download Table S2, XLSX file, 0.1 MB. Copyright © 2016 Galan et al. 2016 Galan et al. This content is distributed under the terms of the Creative Commons Attribution 4.0 International license.

Using mothur v1.34 (42) and the MiSeq standard operating procedure (http://www.mothur.org/wiki/MiSeq_SOP), we removed 20.1% of the paired-end reads because they were misassembled, 1.5% of sequences because they were misaligned, 2.6% because they were chimeric, and 0.2% because they were nonbacterial. The remaining reads were grouped into operational taxonomic units (OTUs) with a divergence threshold of 3%. Bioinformatics analysis identified 13,296 OTUs, corresponding to totals of 7,960,533 sequences in run 1 and 6,687,060 sequences in run 2.

### Taxonomic assignment of sequences.

We used the Bayesian classifier (bootstrap cutoff = 80%) implemented in mothur with Silva SSU Ref database v119 (42) as a reference for the taxonomic assignment of OTUs. The 50 most abundant OTUs accounted for 89% (minimum, 15,284 sequences; maximum, 2,206,731 sequences) of the total sequence data set (see Table S3 in the supplemental material). The accuracy of taxonomic assignment (to the genus level) was assessed with positive controls for PCR, corresponding to DNA extracts from laboratory isolates of *Bartonella taylorii*, *Borrelia burgdorferi*, and *Mycoplasma mycoides* (PC_Bartonella_t_, PC_Borrelia_b_, and PC_Mycoplasma_m_, respectively), which were correctly assigned to a single OTU corresponding to the appropriate reference sequences (Table 2). Note that the sequences of PC_Mycoplasma_m_ were assigned to *Entomoplasmataceae* rather than *Mycoplasmataceae* because of a frequent taxonomic error reflected in most databases, including Silva (43). This problem might also affect other taxa. We therefore recommend systematically carrying out a blast analysis against the sequences of taxa of interest in GenBank to confirm the taxonomic assignment obtained with the 16S databases. Finally, we assumed that the small number of sequences per sample might limit the completeness of bacterial detection (36). For this reason, we discarded seven rodent samples (2 *M. erythroleucus* and 5 *M. domesticus*) yielding fewer than 500 sequences for at least one of the two PCR replicates (1% of the samples).

10.1128/mSystems.00032-16.7 Table S3  The 50 most abundant OTUs in wild rodents and controls. The twelve pathogenic OTUs from wild rodents are indicated in bold and italic. The two OTUs from PC_alien_ (PC_Borrelia_b_ and PC_Mycoplasma_m_) are highlighted in grey. A blank space was added at the end of the table to distinguish the first 50 most abundant OTUs and *Mycoplasma*_OTU_6 and *Rickettsia*_OTU (ranked in position 57 and 574, respectively). Download Table S3, PDF file, 0.1 MB. Copyright © 2016 Galan et al. 2016 Galan et al. This content is distributed under the terms of the Creative Commons Attribution 4.0 International license.

**TABLE 2:** Numbers of sequences for 12 pathogenic OTUs observed in wild rodents, negative controls, and positive controls, together with T_CC_ and T_FA_ threshold values^d^

OTU	Total no. of sequences	Wild rodents (*n* = 711)		Negative controls						Positive controls						Threshold	
				NC_PCR_		NC_ext_		NC_mus_		PC_Bartonnela_t_		PC_Borrelia_b_		PC_Mycoplasma_m_			
		Total no. of sequences	Maximum no. of sequences in one PCR	Total no. of sequences	Maximum no. of sequences in one PCR	Total no. of sequences	Maximum no. of sequences in one PCR	Total no. of sequences	Maximum no. of sequences in one PCR	Total no. of sequences	Maximum no. of sequences in one PCR	Total no. of sequences	Maximum no. of sequences in one PCR	Total no. of sequences	Maximum no. of sequences in one PCR	T_CC_^a^	T_FA_^b^
Run 1																	
Whole data set	7,960,533	7,149,444	64,722	45,900	8,002	39,308	8,741	68,350	26,211	137,424	73,134	239,465	120,552	280,642	82,933	/	/
*Mycoplasma*_OTU_1	1,410,218	1,410,189	61,807	2	1	3	2	9	5	3	3	8	6	4	3	6	282
*Mycoplasma*_OTU_3	507,376	507,369	36,335	2	1	0	0	0	0	2	2	1	1	2	2	2	101
*Ehrlichia*_OTU	649,451	649,423	63,137	4	2	3	2	7	4	1	1	1	1	12	6	6	130
*Borrelia*_OTU	345,873	345,845	28,528	4	4	7	4	9	4	1	1	0	0	7	3	4	69
*Orientia*_OTU	279,965	279,957	29,503	1	1	4	1	0	0	2	2	0	0	1	1	2	56
*Bartonella*_OTU	202,127	67,973	16,145	1	1	1	1	1	1	134,124	71,163	7	4	20	9	9	40
PC*_Mycoplasma_m_*_OTU^c^	280,151	338	28	0	0	0	0	2	2	34	20	24	18	279,753	82,767	/	/
PC*_Borrelia_b_*_OTU^c^	238,772	420	43	0	0	0	0	0	0	38	21	238,238	119,586	76	23	/	/
																	
Run 2																	
Whole data set	6,687,060	6,525,107	42,326	61,231	9,145	53,334	7,669	/	/	12,142	7,518	13,378	7,164	21,868	6,520	/	/
*Mycoplasma*_OTU_1	155,486	155,486	7,703	0	0	0	0	/	/	0	0	0	0	0	0	0	31
*Mycoplasma*_OTU_2	1,036,084	1,035,890	23,588	1	1	192	115	/	/	0	0	0	0	1	1	115	207
*Mycoplasma*_OTU_3	127,591	127,590	5,072	1	1	0	0	/	/	0	0	0	0	0	0	1	26
*Mycoplasma*_OTU_4	85,596	85,583	20,146	0	0	13	13	/	/	0	0	0	0	0	0	13	17
*Mycoplasma*_OTU_5	56,324	56,324	10,760	0	0	0	0	/	/	0	0	0	0	0	0	0	11
*Mycoplasma*_OTU_6	13,356	13,356	1,482	0	0	0	0	/	/	0	0	0	0	0	0	0	3
*Ehrlichia*_OTU	74,017	74,017	19,651	0	0	0	0	/	/	0	0	0	0	0	0	0	15
*Borrelia*_OTU	21,636	21,636	3,085	0	0	0	0	/	/	0	0	0	0	0	0	0	4
*Orientia*_OTU	307	307	181	0	0	0	0	/	/	0	0	0	0	0	0	0	0
*Bartonella*_OTU	1,559,028	1,547,652	14,515	1	1	2	2	/	/	11,297	6,714	2	2	74	59	59	312
*Streptobacillus*_OTU	32,399	32,399	6,245	0	0	0	0	/	/	0	0	0	0	0	0	0	6
*Rickettsia*_OTU	589	589	329	0	0	0	0	/	/	0	0	0	0	0	0	0	0
PC*_Mycoplasma_m_*_OTU^c^	16,854	2	1	0	0	0	0	/	/	0	0	0	0	16,852	5,766	/	/
PC*_Borrelia_b_*_OTU^c^	12,197	0	0	0	0	0	0	/	/	0	0	12,197	6,426	0	0	/	/

a Threshold T_CC_ data are based on the maximum number of sequences observed in a negative or positive control for a particular OTU in each run.

b Threshold T_FA_ data are based on the false-assignment rate (0.02%) weighted by the total number of sequences of each OTU in each run.

c *Mycoplasma mycoides* and *Borrelia burgdorferi* bacterial isolates were added as positive controls for PCR and indexing (i.e., PC_alien_) (see Fig. 1).

d Data are given for the two MiSeq runs separately. NC_PCR_, negative controls for PCR; NC_ext_, negative controls for extraction; NC_mus_, negative controls for dissection; PC_Bartonella_t_, positive controls for PCR; PC_Borrelia_b_ and PC_Mycoplsma_m_, positive controls for PCR and positive controls for indexing; T_CC_ and T_FA_, thresholds for positivity for a particular bacterium according to bacterial OTU and MiSeq run (see also Fig. 1).

### Filtering for reagent contaminants.

**FIG 2:**
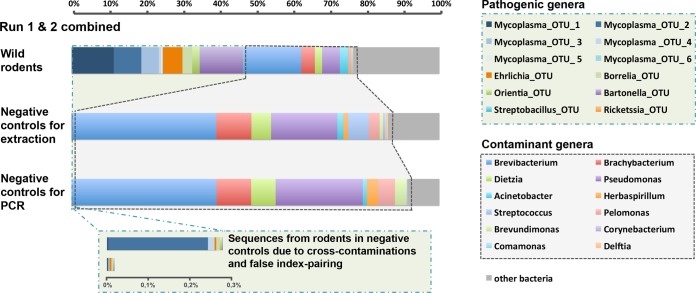
Taxonomic assignment of the V4 16S rRNA sequences in wild rodents and in negative controls for extraction and PCR. The histograms show the percentages of sequences for the most abundant bacterial genera in the two MiSeq runs combined. Notice the presence in the controls of several bacterial genera, which was likely due to the inherent contamination of laboratory reagents by bacterial DNA (termed “contaminant genera”). These contaminant genera are also present (to a lesser extent) in the rodent samples. The insertions represent the proportion of sequences from rodent samples which were incorrectly assigned to the controls. See Fig. S1 for separate histograms for the two MiSeq runs.

16S rRNA amplicon sequencing data may be affected by the contamination of reagents (23). We therefore filtered the data, using negative controls for extraction (NC_ext_), corresponding to extraction without the addition of a tissue sample, and negative controls for PCR (NC_PCR_), corresponding to PCR mixtures to which no DNA was added. We observed between 2,843 and 8,967 sequences in the NC_ext_ and between 5,100 and 9,145 sequences in the NC_PCR_. On the basis of their high number of reads in negative controls, we identified 13 contaminant genera, including *Pseudomonas*, *Acinetobacter*, *Herbaspirillum*, *Streptococcus*, *Pelomonas*, *Brevibacterium*, *Brachybacterium*, *Dietzia*, *Brevundimonas*, *Delftia*, *Comamonas*, *Corynebacterium*, and *Geodermatophilus*, some of them having been previously identified in other studies (23). These contaminants accounted for 29% of the sequences in the data set (Fig. 2). They also differed between MiSeq runs: *Pseudomonas*, *Pelomonas*, and *Herbaspirillum* predominated in run 1, whereas *Brevibacterium*, *Brachybacterium*, and *Dietzia* predominated in run 2 (see Table S4 and Fig. S1 in the supplemental material). This difference probably reflects the use of two different PCR kits manufactured several months apart (Qiagen technical service, personal communication). The majority of the other contaminants, such as *Streptococcus*, most likely originated from the DNA extraction kits used, as they were detected in abundance in the negative controls for extraction (NC_ext_).

10.1128/mSystems.00032-16.1 Figure S1  Taxonomic assignment of the V4 16S rRNA sequences in wild rodents and in negative controls for extraction and of PCR. The histograms show the percentage of sequences for the most abundant bacterial genera in MiSeq run 1 and run 2. Notice the presence of several bacterial genera in the controls, which was likely due to the inherent contamination of laboratory reagents by bacterial DNA and which are therefore called contaminant genera here. These contaminant genera were also present (at lower percentages) in the rodent samples. The differences in bacterial contaminant composition between run 1 and run 2 reflect the use of different kits manufactured several months apart (Qiagen technical service, personal communication). The differences in the proportions and compositions of the pathogenic bacteria between run 1 and run 2 reflect the different origins of the samples as follows. (A) Run 1: *Mastomys erythroleucus* (*n* = 148) and *Mus musculus* (*n* = 207) from northern Senegal. (B) Run 2: *Mastomys erythroleucus* (*n* = 73), *Mastomys natalensis* (*n* = 93), and *Rattus rattus* (*n* = 190) from southern Senegal. Download Figure S1, PDF file, 1.5 MB. Copyright © 2016 Galan et al. 2016 Galan et al. This content is distributed under the terms of the Creative Commons Attribution 4.0 International license.

10.1128/mSystems.00032-16.8 Table S4  Bacterial contaminants observed in negative and positive controls. They were identified as contaminants on the basis of negative controls for extraction and PCR. Taxa indicated in bold correspond to the sequences of DNA extracted from laboratory isolates. Download Table S4, PDF file, 0.1 MB. Copyright © 2016 Galan et al. 2016 Galan et al. This content is distributed under the terms of the Creative Commons Attribution 4.0 International license.

Genera identified as contaminants were then simply removed from the sample data set. Note, however, that the exclusion of these results does not rule out the possibility that our samples represented true rodent infections (at least for some of them, such as those by species of the genus *Streptococcus*, which contains both saprophytic and pathogenic species). However, as mentioned by Razzauti et al. (8), distinguishing between those two possibilities seems difficult, if not impossible. Faced with this lack of certainty, it was most prudent to simply remove these taxa from the sample data set. These results highlight the importance of carrying out systematic negative-control procedures to filter the taxa concerned in order to prevent inappropriate data interpretation, particularly for the *Streptococcus* genus, which contains a number of important pathogenic species. The use of DNA-free reagents would improve the quality of sequencing data and likely increase the depth of sequencing of the samples.

After filtering for reagent contaminants was performed as described above, 12 OTUs, belonging to 7 genera for which at least one species or one strain is known to be pathogenic in mammals (and that are therefore referenced here as “pathogenic genera”), accounted for 66% of the sequences identified in wild-rodent samples for the two MiSeq runs combined (Fig. 2). These genera are *Bartonella*, *Borrelia*, *Ehrlichia*, *Mycoplasma*, *Orientia*, *Rickettsia*, and *Streptobacillus*. Six different OTUs were obtained for *Mycoplasma* (*Mycoplasma*_OTU_1 to *Mycoplasma*_OTU_6) and one OTU each for the other genera (Table 2). Finally, the precise significance of the remaining 34% of sequences, which potentially corresponded to commensal bacteria (*Bacteroidales*, *Bacteroides*, *Enterobacteriaceae*, *Helicobacter*, *Lactobacillus*), unknown pathogens, undetected contaminants, or undetected sequencing errors, was undetermined.

### Filtering for false-positive results.

mothur analysis produced a table of abundance, giving the number of sequences for each OTU in each PCR product (data have been deposited in the Dryad Digital Repository [http://dx.doi.org/10.5061/dryad.m3p7d]) (41). The multiple biases present during the experimental steps and data processing steps listed in Table 1 made it impossible to infer prevalence and cooccurrence directly from the table of sequence presence/absence in the PCR products. We suggest filtering the data with estimates of the different biases calculated from the multiple controls introduced during the process. This strategy involves calculating sequence number thresholds from our bias estimates. Two different thresholds were set for each of the 12 OTUs and two MiSeq runs. We then discarded positive results associated with sequence counts below the threshold (Fig. 1).

### Threshold T_CC_: filtering for cross-contamination.

**FIG 3:**
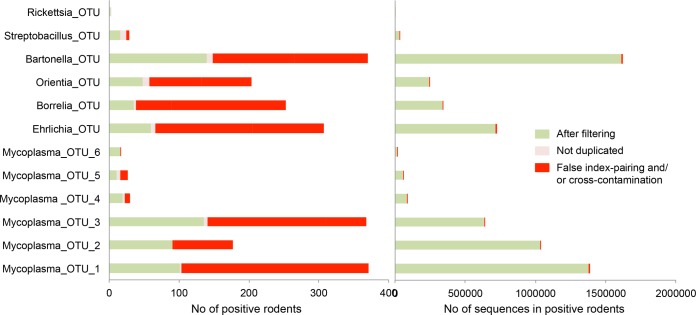
Numbers of rodents yielding positive results (positive rodents), and of sequences from positive rodents, removed for each OTU at each step in data filtering. These findings demonstrate that the positive rodents filtered out corresponded to only a very small number of sequences. (Left panel) The histogram shows the number of positive rodents discarded because of likely cross-contamination, false index pairing, and failure to replicate in both PCRs, as well as the positive results retained at the end of data filtering (in green). (Right panel) The histogram shows the number of sequences corresponding to the same class of positive rodents. Note that several positive results may be recorded for the same rodent in cases of coinfection.

One source of false positives is cross-contamination between samples processed in parallel (Table 1). Negative controls for dissection (NC_mus_), consisting of the spleens of healthy laboratory mice manipulated during sessions of wild-rodent dissection, and negative controls for extraction (NC_ext_) and PCR (NC_PCR_) were used, together with positive controls for PCR (PC_Bartonella_t_, PC_Borrelia_b_, and PC_Mycoplasma_m_), to estimate levels of cross-contamination. For each sequencing run, we calculated the maximal number of sequences for the 12 pathogenic OTUs in the negative and positive controls. These numbers ranged from 0 to 115 sequences, depending on the OTU and the run considered (Table 2), and we used them to establish values for OTU-specific thresholds of cross-contamination (T_CC_) for each run. For example, in sequencing run 2, the highest number of sequences in a control for *Mycoplasma*_OTU_2 was 115 (in an NC_ext_). Therefore, we established the threshold value at 115 sequences for this OTU in sequencing run 2. Thus, PCR products with fewer than 115 sequences for the *Mycoplasma*_OTU_2 in sequencing run 2 were considered to represent false-positive results for this OTU. The use of these T_CC_ values led to 0% to 69% of the positive results being discarded, corresponding to only 0% to 0.14% of the sequences, depending on the OTU considered (Fig. 3; see also Table S5 in the supplemental material). A PCR product may be positive for several bacteria in cases of coinfection. In such cases, the use of a T_CC_ makes it possible to discard the positive result for one bacterium while retaining positive results for other bacteria.

10.1128/mSystems.00032-16.9 Table S5  Proportion of sequences and proportion of positive results removed at each step in data filtering. Note that several positive results may be recorded for the same rodent in cases of coinfection. Download Table S5, PDF file, 0.1 MB. Copyright © 2016 Galan et al. 2016 Galan et al. This content is distributed under the terms of the Creative Commons Attribution 4.0 International license.

### Threshold T_FA_: filtering out incorrectly assigned sequences.

Another source of false positives is the incorrect assignment of sequences to a PCR product (Table 1). This phenomenon may be due either to cross-contamination between indices during the experiment or to the generation of mixed clusters during the sequencing (27).

First, the cross-contamination of indices may happen during the preparation of indexed primer microplates. This cross-contamination was estimated using negative-control index pairs (NC_index_) corresponding to particular index pairs not used to identify the samples. NC_index_ returned very low (1 to 12) read numbers, suggesting that there was little or no cross-contamination between indices in our experiment.

Second, the occurrence of mixed clusters during the sequencing of multiplexed samples was previously reported by Kircher et al. (27). Mixed clusters on an Illumina flow cell surface are described by Kircher et al. (27) as the predominant source of error of sequence assignment to a PCR product. The impact of this phenomenon on our experiment was estimated using “alien” positive controls (PC_alien_) (sequenced in parallel with the rodent samples): PC_Mycoplasma_m_, corresponding to the DNA of *Mycoplasma mycoides*, which cannot infect rodents; and PC_Borrelia_b_, containing the DNA of *Borrelia burgdorferi*, which is not present in Africa. Neither of these bacterial species can survive in abiotic environments, so the presence of their sequences in African rodent PCR products indicates a sequence assignment error due to false index pairing (27). Using PC_Mycoplasma_m_, we obtained an estimate of the global false-index-pairing rate of 0.14% (i.e., 398 of 280,151 sequences of the *Mycoplasma mycoides* OTU were assigned to samples other than PC_Mycoplasma_m_). Using PC_Borrelia_b_, we obtained an estimate of 0.22% (534 of 238,772 sequences of the *Borrelia burgdorferi* OTU were assigned to samples other than PC_Borrelia_b_). These values are very close to the estimate of 0.3% obtained by Kircher et al. (27). Close examination of the distribution of misassigned sequences within the PCR 96-well microplates showed that all PCR products with misassigned sequences had one index in common with either PC_Mycoplasma_m_ or PC_Borrelia_b_ (see Fig. S2 in the supplemental material).

10.1128/mSystems.00032-16.2 Figure S2  Numbers of sequences of the positive controls used for indexing PC_Borrelia_b_ (in blue) and PC_Mycoplasma_m_ (in red) in the various PCR products, with a dual-indexing design, for MiSeq run 1 (a) and run 2 (b). The two PCRs for PC_Borrelia_b_ were performed with 96-well microplate 9 and positions A1 and E1 for run 1 and B1 and F1 for run 2, and the four PCRs for PC_Mycoplasma_m_ were performed with 96-well microplate 9 and positions C1, D1, G1, and H1 for the two runs. The numbers of sequences for the other wells correspond to indexing mistakes due to false index pairing due to the presence of mixed clusters during the sequencing (see Table 1). Download Figure S2, PDF file, 0.4 MB. Copyright © 2016 Galan et al. 2016 Galan et al. This content is distributed under the terms of the Creative Commons Attribution 4.0 International license.

We then estimated the impact of false index pairing for each PCR product by calculating the maximal number of sequences of alien bacteria assigned to PCR products other than the corresponding PC. These numbers ranged from 28 to 43, depending on the positive control for run 1 (Table 2)—run 2 was discarded because of the low values of the numbers of sequences, which were obtained likely due to the fact that DNAs of PC were diluted 100-fold in run 2 (see Table S1 in the supplemental material). We then estimated a false-assignment rate for each PCR product (*R*_fa_) by dividing the numbers given above by the total number of sequences from alien bacteria in sequencing run 1. *R*_fa_ was estimated for PC_Mycoplasma_m_ and PC_Borrelia_b_ separately. *R*_fa_ reached 0.010% and 0.018% for PC_Mycoplasma_m_ and PC_Borrelia_b_, respectively. We adopted a conservative approach by fixing the *R*_fa_ value at 0.020%. This number signifies that each PCR product may receive a maximum of 0.020% of the total number of sequences of an OTU present in a run due to false index pairing. Moreover, the number of sequences for a specific OTU misassigned to a PCR product should increase with the total number of sequences of the OTU in the MiSeq run. We therefore defined the second threshold (T_FA_) as the total number of sequences in the run for an OTU multiplied by *R*_fa_. T_FA_ values varied with the abundance of each OTU in the sequencing run (Table 2). Because the abundance of each OTU varied from one sequencing run to the next, T_FA_ also varied according to the sequencing run. The use of the T_FA_ led to 0% to 87% of positive results being discarded. This corresponded to 0% to 0.71% of the sequences, depending on the OTU (Fig. 3; see also Table S5 in the supplemental material).

### Validation performed with PCR replicates.

**FIG 4:**
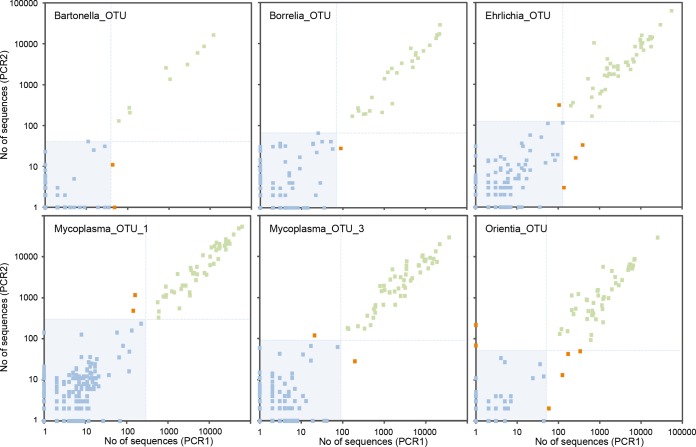
Plots of the number of sequences [log (*x* + 1) scale] from bacterial OTUs in both PCR replicates (PCR1 and PCR2) of the 348 wild rodents analyzed in the first MiSeq run. Note that each rodent was tested with two replicate PCRs. Green points correspond to rodents with two positive results after filtering; red points correspond to rodents with one positive result and one negative result; and blue points correspond to rodents with two negative results. The light blue area and lines correspond to threshold values used for the data filtering: samples below the lines were filtered out. See Fig. S3 for plots corresponding to the second MiSeq run.

Random contamination may occur during the preparation of PCR 96-well microplates. These contaminants may affect some of the wells, but not those for the negative controls, leading to the generation of false-positive results. We thus adopted a conservative approach in which we considered rodents to be positive for a given OTU only if both PCR replicates were considered to represent positive results after the filtering steps described above were performed. The relevance of this strategy was supported by the strong correlation between the numbers of sequences for the two PCR replicates for each rodent (*R*^2^ > 0.90) (Fig. 4; see also Fig. S3 in the supplemental material). At this stage, 673 positive results for 419 rodents were validated for both replicates (note that a rodent sample may be positive for several bacterial species and may thus be counted several times), whereas only 52 positive results were discarded because the result for the other replicate was negative. At this final validation step, 0% to 60% of the positive results for a given OTU were discarded, corresponding to only 0% to 7.17% of the sequences (Fig. 3; see also Table S5 and Table S6 in the supplemental material). Note that the number of replicates may be increased, as previously described for the strategy of Gómez-Díaz et al (44).

10.1128/mSystems.00032-16.3 Figure S3  Plots of the number of sequences [log (*x* + 1) scale] from bacterial OTUs in both PCR replicates (PCR1 and PCR2) for the 356 wild rodents analyzed in the second MiSeq run. Note that each rodent was tested with two replicate PCRs. Green points correspond to rodents with two positive results after the filtering process; red points correspond to rodents with one positive result and one negative result; and blue points correspond to rodents with two negative results. The light blue area and lines correspond to the threshold values used for the data filtering: samples below the lines were filtered out. See Fig. 4 for plots corresponding to the first MiSeq run. Download Figure S3, PDF file, 0.1 MB. Copyright © 2016 Galan et al. 2016 Galan et al. This content is distributed under the terms of the Creative Commons Attribution 4.0 International license.

10.1128/mSystems.00032-16.10 Table S6  Proportion of positive results for both PCR products at each step in data filtering. Note that several positive results may have been recorded for the same rodent in cases of coinfection. Download Table S6, PDF file, 0.1 MB. Copyright © 2016 Galan et al. 2016 Galan et al. This content is distributed under the terms of the Creative Commons Attribution 4.0 International license.

### Postfiltering results.

Finally, the proportions of rodents positive for a given OTU filtered out by the complete filtering approach ranged from 6% to 86%, depending on the OTU, corresponding to only 1% of the total sequences (Fig. 3). Indeed, our filtering strategy mostly excluded rodents with a small number of sequences for the OTU concerned. These rodents were considered to represent false-positive results.

### Refining bacterial taxonomic identification.

We refined the taxonomic identification of the 12 bacterial OTUs through phylogenetic and blast analyses. We were able to identify the bacteria present down to the genus level, and, in some cases, we could even identify the most likely species (Table 3; see also Fig. S4 in the supplemental material). For instance, the sequences of the six *Mycoplasma* OTUs were consistent with three different species—*M. haemomuris* for OTU_1 and OTU_3, *M. coccoides* for OTU_4, OTU_5, and OTU_6, and *M.* sp. nov. (45) for OTU_2—with high percentages of sequence identity (≥93%) and bootstrap values (≥80%). All three of these species belong to the *Hemoplasma* group, whose members are known to infect mice, rats, and other mammals (46, 47) and are thought to cause anemia in humans (48, 49). The *Borrelia* sequences grouped with three different species of the relapsing fever group (*B. crocidurae*, *B. duttonii*, and *B. recurrentis*) with a high percentage of identity (100%) and a bootstrap value of 71%. In West Africa, *B. crocidurae* causes severe borreliosis, a rodent-borne disease transmitted by ticks and lice (50). The *Ehrlichia* sequences were 100% identical to and clustered with the sequences of the recently described “*Candidatus* Ehrlichia khabarensis” species isolated from voles and shrews in the Far East of the Russian Federation (51). The *Rickettsia* sequences were 100% identical to the sequence of *R. typhi*, a species of the typhus group responsible for murine typhus (52), but species of this clade were differentiated from many other *Rickettsia* species only weakly (bootstrap support of 61%). The most likely species corresponding to the sequences of the *Streptobacillus* OTU was *S. moniliformis*, with a high percentage of identity (100%) and a bootstrap value of 100%. This bacterium is common in rats and mice and causes a form of rat-bite fever, Haverhill fever (53). The *Orientia* sequences corresponded to *O. chuto*, with a high percentage of identity (100%) and a bootstrap value of 77%. This species was recently isolated from a patient infected in Dubai (54). Finally, accurate species determination was not possible for *Bartonella*, as the 16S rRNA gene does not resolve the species of this genus well (55). Indeed, the sequences from the *Bartonella* OTU detected in our rodents corresponded to at least seven different species (*B. elizabethae*, *B. japonica*, *B. pachyuromydis*, *B. queenslandis*, *B. rattaustraliani*, *B. tribocorum*, and *B. vinsonii*) and a putative new species recently identified in Senegalese rodents (56).

10.1128/mSystems.00032-16.4 Figure S4  Phylogenetic trees of the 16S rRNA V4 sequences for 12 pathogenic bacterial OTUs detected in wild rodents from Senegal. Sequences boxed with an orange line were retrieved from African rodents and/or correspond to positive controls (PC) for *Borrelia burgdorferi*, *Mycoplasma mycoides*, and *Bartonella taylorii*. The other sequences were extracted from the SILVA database and GenBank. Trees include all lineages collected for *Rickettsia*, *Bartonella*, *Ehrlichia*, and *Orientia*, but only lineages of the spotted fever group for *Borrelia* and lineages of the pneumonia group for *Mycoplasma*. The numbers indicated are the bootstrap values of >55%. The Fasta files used have been deposited in the Dryad Digital Repository (http://dx.doi.org/10.5061/dryad.m3p7d). Download Figure S4, PDF file, 0.2 MB. Copyright © 2016 Galan et al. 2016 Galan et al. This content is distributed under the terms of the Creative Commons Attribution 4.0 International license.

**TABLE 3:** Detection of 12 bacterial OTUs in the four wild-rodent species sampled in Senegal: biology and pathogenicity of the corresponding bacterial genus

OTU of interest (genus level)	Closest species^a^ **(% identity in GenBank)**	No. of positive wild rodents (*n* = 704)^b^				Biology and epidemiology
		*Mastomys erythroleucus* (*n* = 219)	*Mastomys natalensis* (*n* = 93)	*Mus musculus* (*n* = 203)	*Rattus rattus* (*n* = 189)	
*Bartonella*	Undetermined	60	73	1	6	*Bartonella* spp. are intracellular fastidious hemotropic Gram-negative organisms identified in a wide range of domestic and wild mammals and transmitted by arthropods. Several rodent-borne *Bartonella* species have emerged as zoonotic agents, and various clinical manifestations are reported, including fever, bacteremia and neurological symptoms (82).
*Borrelia*	*B. crocidurae* (100)	21	0	8	6	*Borrelia* is a genus of spiral Gram-negative bacteria of the spirochete phylum. These bacteria are obligate parasites of animals and are responsible for relapsing fever borreliosis, a zoonotic disease transmitted by arthropods (ticks and lice). This disease is the most frequent human bacterial disease in Africa. West Africa, including Senegal, is a region of endemicity for disease caused by *B. crocidurae*, and *B. duttonii* and *B. recurrentis* have been reported in Central, southern and East Africa (50).
	*B. duttonii* (100)					
	*B. recurrentis* (100)					
*Ehrlichia*	“*Ca.* Ehrlichia khabarensis” (100)	40	0	12	8	The genus *Ehrlichia* includes five species of small Gram-negative obligate intracellular bacteria. The life cycle includes the reproduction stages taking place in both ixodid ticks, acting as vectors, and vertebrates. *Ehrlichia* spp. can cause a persistent infection in the vertebrate hosts, which thus become reservoirs of infection. A number of new genetic variants of *Ehrlichia* have been recently detected in rodent species (e.g., “*Ca.* Ehrlichia khabarensis”) (51).
*Mycoplasma*_Otu_1	*M.* *haemomuris* (96)	28	42	30	1	*Mycoplasma* is a genus that includes over 100 species of bacteria that lack of a cell wall around their cell membrane. *Mycoplasma coccoides* and *Mycoplasma haemomuris* are blood parasites of wild and laboratory rodents. A new closely related species (AB752303) was recently isolated from brown rats (45). These species are commonly referred as “hemoplasmas.” Hemoplasmas have been detected within the erythrocytes of cats, dogs, pigs, rodents, and cattle, in which they may cause anemia. There have been sporadic reports of similar infections in humans, but these infections have been poorly characterized (49).
*Mycoplasma*_Otu_2	*M.* sp. nov. (100) (GenBank accession no. AB752303)	0	0	0	90	
*Mycoplasma*_Otu_3	*M.* *haemomuris* (93)	93	40	1	1	
*Mycoplasma*_Otu_4	*M.* *coccoides* (96)	0	0	0	18	
*Mycoplasma*_Otu_5	*M.* *coccoides* (95)	3	8	0	0	
*Mycoplasma*_Otu_6	*M.* *coccoides* (97)	3	13	0	0	
*Orientia*	*O. chuto* (100)	0	2	46	0	*Orientia* is a genus of obligate intracellular Gram-negative bacteria found in mites and rodents. *Orientia tsutsugamushi* is the agent of scrub typhus in humans. This disease, one of the most underdiagnosed and underreported febrile illnesses requiring hospitalization, has an estimated 10% fatality rate unless treated appropriately. A new species, *Orientia chuto*, was recently characterized in sick patients from the Arabian Peninsula, and new *Orientia* haplotypes have been identified in France and Senegal (9).
	*O. tsutsugamushi* (98)					
*Rickettsia*	*R. typhi* (100)	1	0	0	1	*Rickettsia* is a genus of obligate intracellular Gram-negative bacteria found in arthropods and vertebrates. *Rickettsia* spp. are symbiotic species transmitted vertically in invertebrates, and some are pathogenic invertebrates. Infections by *Rickettsia* species of the typhus group result in many human diseases, including murine typhus, which is caused by *Rickettsia typhi* and transmitted by fleas (52).
*Streptobacillus*	*S. moniliformis* (100)	10	1	0	5	*Streptobacillus* is a genus of aerobic, Gram-negative facultative anaerobe bacteria, which grow in culture as rods in chains. *Streptobacillus moniliformis* is common in rats and mice and is responsible of the streptobacillosis form of rat-bite fever, the Haverhill fever. This zoonosis begins with high prostrating fevers, rigors (shivering), headache, and polyarthralgia (joint pain). Left untreated, rat-bite fever has a mortality rate of approximately 10% (53).

a Based on phylogenetic analysis; see Fig. S4 in the supplemental material.

b *n*, number of rodents screened and analyzed.

These findings demonstrate the considerable potential of 16S rRNA amplicon sequencing for the rapid identification of zoonotic agents in wildlife, provided that the postsequencing data are cleaned beforehand. *Borrelia* (50) and *Bartonella* (56) were the only ones of the seven pathogenic bacterial genera detected here in Senegalese rodents to have been reported as present in rodents from West Africa before. The other bacterial genera identified here have previously been reported to be present in rodents only in other parts of Africa or on other continents. *Streptobacillus moniliformis* has recently been detected in rodents from South Africa (57), and there have been a few reports of human streptobacillosis in Kenya (58) and Nigeria (59). *R. typhi* was recently detected in rats found in Zaire, in Central Africa (60), and human seropositivity for this bacterium has been reported in coastal regions of West Africa (61). With the exception of one study in Egypt published some time ago (62), *Mycoplasma* spp. have never before been reported in African rodents. Several species of *Ehrlichia* (from the *E. canis* group: *E. chaffeensis*, *E. ruminantium*, *E. muris*, and *E. ewingii*) have been characterized in West Africa, but only in ticks from cattle (63), and a report of possible cases of human ehrlichioses in this region was previously published (64). Finally, the present study reports the first identification of *Orientia* in African rodents (9). There have already been a few reports of suspected human infection with this bacterium in Zaire, Cameroon, Kenya, and Tanzania (65).

### Estimating prevalence and coinfection.

After performing data filtering, we were able to estimate the prevalence in rodent populations and to assess coinfection in individual rodents for the 12 bacterial OTUs. Rates of bacterial prevalence differed considerably between rodent species (Table 3). *Bartonella* spp. were highly prevalent in the two multimammate rat species *M. natalensis* (79%) and *M. erythroleucus* (27%); *Orientia* spp. were prevalent in the house mouse species *M. musculus* (22%); and *Ehrlichia* spp. occurred frequently in only one of the two rats of the multimammate species *M. erythroleucus* (18%). In contrast, the prevalence of *Streptobacillus* and *Rickettsia* was low in all rodent species (<5%). Coinfection was common, as 184 rodents (26%) were found to be coinfected with bacteria from two (19%), three (5%), four (2%), or five (0.1%) different bacterial pathogens.

**FIG 5:**
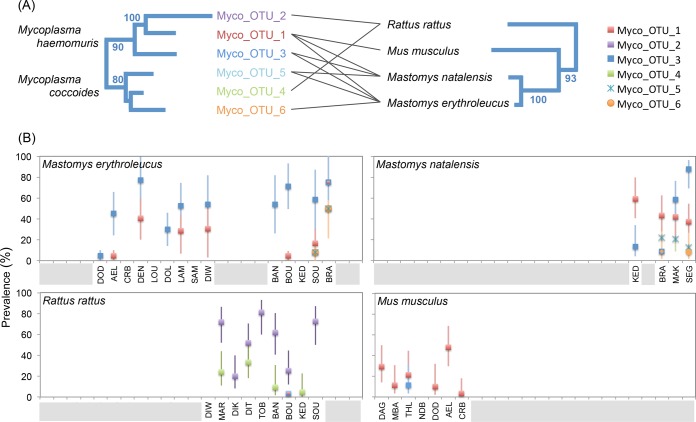
Prevalence of *Mycoplasma* lineages in Senegalese rodents, by site, and phylogenetic associations between *Mycoplasma* lineages and rodent species. (A) Comparison of phylogenetic trees based on the 16S rRNA V4 sequences of *Mycoplasma* and on the mitochondrial cytochrome *b* gene and the two nuclear gene fragments (IRBP exon 1 and GHR) for rodents (the tree was drawn based on data from reference 92). Lines link the *Mycoplasma* lineages detected in the various rodent species (for a minimum site prevalence exceeding 10%). The numbers next to the branches are bootstrap values (shown only if >70%). (B) Plots of OTU prevalences, with 95% confidence intervals calculated by Sterne’s exact method (93) according to rodent species and site (see reference 69 for more information about site codes and their geographic locations). The gray bars on the *x* axis indicate sites from which the rodent species concerned is absent.

Interestingly, several *Mycoplasma* OTUs appeared to be specific to a rodent genus or species (Table 3 and Fig. 5A). OTU_2, putatively identified as a recently described lineage isolated from brown rat, *Rattus norvegicus* (45), was specifically associated with *R. rattus* in this study. Of the OTUs related to *M. coccoides*, OTU_4 was found exclusively in *R. rattus*, whereas OTU_5 and OTU_6 seemed to be specific to the two multimammate rats (*M. erythroleucus and M. natalensis*). Comparative phylogenies of *Mycoplasma* OTUs and rodents showed that *R. rattus*, which is phylogenetically more distantly related to the other three rodents, contained a *Mycoplasma* community different from that in the rodents in the *Mus*-*Mastomys* clade (Fig. 5A). Pathogen prevalences also differed considerably between sites, as shown for the six *Mycoplasma* OTUs (Fig. 5B). This suggests that the infection risks for animals and humans vary greatly according to environmental characteristics and/or biotic features potentially related to recent changes in the distribution of rodent species in Senegal (66, 67).

### Perspectives. (i) Recommendation for future experiments.

Our experiments demonstrated the need to include many different kinds of controls, at different steps, in order to avoid data misinterpretation. In particular, alien positive controls are important for establishing threshold values for OTU positivity. These alien positive controls should include taxa that are distant enough from those of potential pathogens to avoid potential confusion between sequences of alien controls and sequences that result from actual infection of rodent samples. Ideally, one should choose alien positive controls from bacterial genera which are not able to infect the study’s host species. In our study, the use of *Mycoplasma* and *Borrelia* species as alien positive controls was not ideal because both genera are potential rodent pathogens. Thankfully, the species used as alien controls could be easily distinguished from the species found in rodents on the basis of the phylogenetic analyses of the V4 sequences. However, on the basis of our experience, we recommend using bacterial genera phylogenetically distant from pathogenic genera as alien controls when possible.

The inclusion of negative controls of PCR and DNA extraction in studies based on massive sequencing of 16S rRNA amplicons had long been overlooked until a report published by Salter in 2014 (23) demonstrated the high pollution of laboratory reagents by bacterial DNA. Most studies published prior to that report reported no extraction controls in their protocols. Here, we performed one negative control for extraction per DNA extraction microplate; with each run consisting of four DNA extraction microplates, and each control having been analyzed in two replicate experiments, we had a total of 8 negative controls for extraction per run which were analyzed twice. On the basis of our experience, we recommend performing at least this number of extraction controls per run. Further increases in the number of extraction controls per microplate would further improve the efficiency of data filtering and so the quality of the data produced.

The protocol of PCR amplification is also of importance for ensuring data quality. In our study, we built separate amplicon libraries for each sample separately and used very long (5-min) PCR elongation times in order to mitigate the formation of chimeric reads (18). High numbers of PCR cycles are also known to increase chimera formation, yet, as mentioned by Schnell et al. (68), this parameter is mainly critical only when bulk amplification of pools of tagged/indexed amplicons is performed (e.g., when using an Illumina TrueSeq library preparation kit). As we used separate amplicon libraries for each sample, we believe that the relatively high number of PCR cycles that we used (40 cycles) had minimal impact on chimera formation and that this protocol ensures the absence of chimeric sequences between samples. We had chosen to maximize the number of cycles to enhance our ability to detect pathogenic bacteria, which are sometimes of low quantity in animal samples. Fine-tuning the balance between these parameters deserves further study.

Moreover, we targeted the spleen to detect bacterial infections in our study based on the fact that this organ is known to filter microbial cells in mammals. However, we lack the data to be certain that the spleen is the best organ for use in giving a global picture of bacterial infection in rodents (and, more broadly, in vertebrates). We are currently conducting new experiments to address this issue.

Finally, in our experiments, about a third of OTU sequences were attributable neither to contamination nor to (known) pathogenic genera. We currently have no precise idea of the significance of the presence of these OTUs in the rodent spleens. Some of these OTUs could be linked to further undetected biases during data generation; in spite of all the precautions we have implemented here, other biases may still elude detection. Such biases could explain the very high numbers of rare OTUs (11,947 OTUs corresponding to <100 reads), which together represent more than 88% of the total number of OTUs but less than 1% of the total number of sequences (with the two runs combined).

Additionally, the presence of an OTU in a rodent spleen does not necessarily imply that the OTU is pathogenic. We know little about the microbiome of healthy organs of vertebrates, and yet the sharp increase in the number of microbiome studies over the last few years has led to the discovery that communities of microbiota appear to be specific to each part of the vertebrate’s body, including internal tissues and blood (69). The OTUs detected in rodent’s spleen could thus simply be part of the healthy microbiome of the organ. These issues deserve better documentation. Our results thus pave the way for future research on unknown bacterial pathogens and on the microbiome of healthy organs in vertebrates.

### (ii) Improving HTS for epidemiological surveillance.

The screening strategy described here has the considerable advantage of being nonspecific, making it possible to detect unanticipated or novel bacteria. Razzauti et al. (8) recently showed that the sensitivity of 16S rRNA amplicon sequencing on the MiSeq platform was equivalent to that of whole-RNA sequencing (RNA-seq) on the HiSeq platform for detecting bacteria in rodent samples. However, little is known about the comparative sensitivities of HTS approaches relative to the sensitivity of quantitative PCR (qPCR) performed with specific primers, the current gold standard for bacterial detection within biological samples. Additional studies are required to address this issue. Moreover, as 16S rRNA amplicon sequencing is based on a short sequence, it does not yield results sufficiently high in resolution to distinguish between species in some bacterial genera, such as *Bartonella*, or to distinguish between pathogenic and nonpathogenic strains within the same bacterial species. To get this information, we thus need to follow up the 16S rRNA amplicon sequencing with complementary laboratory work. Whole-genome shotgun or RNA-seq techniques provide longer sequences, through the production of longer reads or the assembly of contigs, and they might therefore increase the accuracy of species detection (70). However, these techniques would be harder to adapt for the extensive multiplexing of samples (8). Other methods could be used to assign sequences to bacterial species or strains for samples found to be positive for a bacterial genus following the 16S rRNA screening. For example, positive PCR assays could be carried out with bacterial genus-specific primers, followed by amplicon sequencing, as commonly used in multilocus sequence analysis (MLSA) strategies (71), or high-throughput microfluidic qPCR assays based on bacterial species-specific primers could be used (72). High-throughput amplicon sequencing approaches could be fine-tuned to amplify several genes for species-level assignment, such as the *gltA* gene used by Gutiérrez et al. (73) for the *Bartonella* genus, in parallel with the 16S rRNA-V4 region.

This strategy could also easily be adapted for other microbes, such as protists, fungi, and even viruses, provided that universal primers are available for their detection (see references 74 and 75 for protists and fungi and reference 76 for degenerate virus family-level primers for viruses). Finally, our filtering method could also be translated to any other postsequencing data set of indexed or tagged amplicons in the framework of environmental studies (e.g., metabarcoding for diet analysis and biodiversity monitoring [77], the detection of rare somatic mutations [78], or the genotyping of highly polymorphic genes [e.g., major histocompatibility complex {MHC} or HLA typing] [79, 80]).

### (iii) Monitoring the risk of zoonotic diseases.

Highly successful synanthropic wildlife species, such as the rodents studied here, will probably play an increasingly important role in the transmission of zoonotic diseases (81). Many rodent-borne pathogens cause only mild or undifferentiated disease in healthy people, and these illnesses are often misdiagnosed and underreported (53, 82–85). The information about pathogen circulation and transmission risks in West Africa provided by this study is important in terms of human health policy. We show that rodents carry species of seven major pathogenic bacterial genera: *Borrelia*, *Bartonella*, *Mycoplasma*, *Ehrlichia*, *Rickettsia*, *Streptobacillus*, and *Orientia*. The last five of these genera have never before been reported in West African rodents. The data generated with our HTS approach could also be used to assess zoonotic risks and to formulate appropriate public health strategies involving the focusing of continued pathogen surveillance and disease-monitoring programs on specific geographic areas or rodent species likely to be involved in zoonotic pathogen circulation, for example.

## MATERIALS AND METHODS

### Ethics statement.

Animals were treated in accordance with European Union guidelines and legislation (Directive 86/609/EEC). The CBGP laboratory received approval (no. B 34-169-003) from the Departmental Direction of Population Protection (DDPP, Hérault, France), for the sampling of rodents and the storage and use of their tissues. None of the rodent species investigated in this study has protected status (see the International Union for the Conservation of Nature [IUCN] and Convention on International Trade in Endangered Species of Wild Flora and Fauna [CITES] lists).

### Experimental controls.

Recent research has highlighted different biases occurring at different steps of high-throughput sequencing. These biases can be estimated directly from the data by including several controls together with samples in the experiment. We detail below these different controls as well as the rationale for their use.

### (i) Negative controls for sample collection.

When possible, we advise inclusion of axenic samples during collection of the samples. The numbers of sequences observed in these controls are used to estimate cross-contamination rates during sample collection. In our study, we used spleens from healthy laboratory mice (NC_mus_), free from rodent pathogens, which were manipulated together with wild-rodent samples during the dissections in the field.

### (ii) Negative controls for DNA extraction (NC_ext_).

DNA extractions should be performed without the addition of sample tissue (blanks), which are processed together with the other samples. We advise performing at least one extraction blank experiment for each extraction experiment, although more would be better. The numbers of sequences observed in these controls are used to estimate and filter the cross-contaminations during the DNA extractions and to detect any DNA bacterial contaminants present in the extraction kit reagents.

### (iii) Negative controls for PCR (NC_PCR_).

PCRs should be performed without any DNA extract included (blank), which should be processed together with the other samples. We advise performing at least one PCR blank experiment per PCR microplate, although more would be better. The numbers of sequences observed in these controls are used to estimate and filter the cross-contaminations during the PCR preparation and to detect any DNA bacterial contaminants present in the PCR reagents.

### (iv) Negative controls for indexing (NC_index_).

Combinations of barcodes that are not used to identify samples in the sequencing run should be searched for during the bioinformatic demultiplexing. In practice, they should correspond to empty PCR wells (without reagent and without index). The numbers of sequences recovered for these particular index combinations should be used to estimate and filter the cross-contaminations between indexed PCR primers during primer handling or PCR preparation and to identify errors in an Illumina sample sheet.

### (v) Positive controls for PCR (PC_PCR_).

DNA from isolates of known taxa should be processed together with the other samples. The sequences obtained for these controls should be used to verify the taxonomic assignment and to estimate and filter cross-contaminations.

### (vi) Positive controls for indexing (PC_alien_).

DNA from isolates of known taxa should be absent from the samples. They should be handled separately from the samples to avoid cross-contaminations with the samples during the wet laboratory procedures (DNA extractions and PCRs). Sequences from PC_alien_ found in the samples are used to calculate the rate of sample misidentification due to false index pairing (see text and Kircher et al. [27] for details concerning this phenomenon).

In practice, PC_PCR_ and PC_alien_ could be the same, and we advise the use of taxa that are phylogenetically distant from the taxa of interest in order to avoid potential confusion between sequences from alien controls and sequences from the samples.

### Sample collection.

We sampled rodents in 24 villages of the Sahelian and Sudanian climatic and biogeographical zones in Senegal (see Dalecky et al. [67] for details on the geographic location and other information on the villages). Rodents were sampled by live trapping according to the standardized protocol described by Dalecky et al. (67). Briefly, traps were set within homes (with one single-capture wire-mesh trap and one Sherman folding box trap used per room) during one day to five consecutive days. Each captured rodent was collected alive and transported to the field laboratory. There, rodents were killed by cervical dislocation as recommended by Mills et al. (86) and dissected as described by Herbreteau et al. (87). Rodent species were identified by morphological and/or molecular techniques (67). The information concerning the rodent collection (sample identifier [ID], locality, and species) is provided in Table S2 in the supplemental material. Cross-contamination during dissection was prevented by washing the tools used successively in bleach, water, and alcohol between rodent procedures. We used the spleen for bacterial detection, because this organ is a crucial site of early exposure to bacteria (88). Spleens were placed in RNAlater (Sigma) and stored at 4°C for 24 h and then at −20°C until their use for genetic analyses.

### Target DNA region and primer design.

We used primers with sequences slightly modified from those of the universal primers described by Kozich et al. (18) to amplify a 251-bp portion of the V4 region of the 16S rRNA gene (16S-V4F [GTGCCAGCMGCCGCGGTAA] and 16S-V4R [GGACTACHVGGGTWTCTAATCC]). The ability of these primers to hybridize to the DNA of bacterial zoonotic pathogens was assessed by checking that there were low numbers of mismatched bases over an alignment of 41,113 sequences from 79 zoonotic genera inventoried by Taylor et al. (1), extracted from Silva SSU database v119 (89). The FASTA file has been deposited in the Dryad Digital Repository (http://dx.doi.org/10.5061/dryad.m3p7d) (41).

We used a slightly modified version of the dual-index method of Kozich et al. (18) to multiplex our samples. The V4 primers included different 8-bp indices (called the i5 index in the forward primer and the i7 index in the reverse primer) and Illumina adapters (called the P5 adapter in the forward primer and the P7 adapter in the reverse primer) in the 5′ position. The combinations of 24 i5-indexed primers and 36 i7-indexed primers made it possible to identify 864 different PCR products loaded onto the same MiSeq flow cell. Each index sequence differed from the others by at least two nucleotides, and each nucleotide position in the sets of indices contained approximately 25% of each base, to prevent problems due to Illumina low-diversity libraries (Table 1).

### DNA extraction and PCRs.

All pre-PCR laboratory manipulations were conducted with filter tips under a sterile hood in a DNA-free room, i.e., a room dedicated to the preparation of PCR mix and equipped with hoods that are kept free of DNA by UV irradiation and bleach treatment. DNA from bacterial isolates (corresponding to DNA extracts from laboratory isolates of *Bartonella taylorii*, *Borrelia burgdorferi*, and *Mycoplasma mycoides*) was extracted in another laboratory, and PCRs using these isolates were performed after the amplifications of the DNA from rodents to avoid cross-contamination between samples and bacterial isolates. DNA was extracted with a DNeasy 96 tissue kit (Qiagen), with final elution in 200 µl of elution buffer. One extraction blank (NC_ext_), corresponding to an extraction without sample tissue, was systematically added to each of the eight DNA extraction microplates. DNA was quantified with a NanoDrop 8000 spectrophotometer (Thermo Scientific) to confirm the presence of a minimum of 10 ng/µl of DNA in each sample. DNA amplification was performed in 5 µl of Multiplex PCR kit (Qiagen) master mix, with 4 µl of combined i5 and i7 primers (3.5 µM) and 2 µl of genomic DNA. The PCR began with an initial denaturation at 95°C for 15 min; followed by 40 cycles of denaturation at 95°C for 20 s, annealing at 55°C for 15 s, and extension at 72°C for 5 min; followed by a final extension step at 72°C for 10 min. PCR products (3 µl) were verified by electrophoresis in a 1.5% agarose gel. One PCR blank (NC_PCR_), corresponding to the PCR mix with no DNA, was systematically added to each of the 18 PCR microplates. DNA was amplified in replicate for all wild-rodent samples (*n* = 711) (for a summary, see Table S1 in the supplemental material; for details by sample, see Table S2).

### Library preparation and MiSeq sequencing.

Two Illumina MiSeq runs were conducted. Run 1 included the PCR products (two or three replicates per sample) from wild rodents collected in north Senegal (148 *Mastomys erythroleucus* and 207 *Mus musculus*) plus the positive controls and the negative controls. Run 2 included the PCR products (two replicates per samples) from wild rodents collected in south Senegal (73 *Mastomys erythroleucus*, 93 *Mastomys natalensis*, and 190 *Rattus rattus*) plus the positive controls and the negative controls. Full details on the composition of runs are given in Table S2 in the supplemental material. The MiSeq platform was chosen because it generates lower error rates than other HTS platforms (90). The numbers of PCR products multiplexed were 823 for the first MiSeq run and 746 for the second MiSeq run (see Table S2). Additional PCR products from other projects were added to give a total of 864 PCR products per run. PCR products were pooled by volume for each 96-well PCR microplate, using 4 µl for rodents and controls and 1.5 µl for bacterial isolates. Mixes were checked by electrophoresis on 1.5% agarose gels before their use to generate a “superpool” of 864 PCR products for each MiSeq run. We subjected 100 µl of each superpool to size selection for the full-length amplicon (expected median sizes of V4 hypervariable region, 375 bp [including primers, indices, and adaptors] and 251 bp [excluding primers, indices, and adaptors]) by excision from a low-melting (1.25%) agarose gel to discard nonspecific amplicons and primer dimers. A PCR cleanup gel extraction kit (Macherey-Nagel) was used to purify the excised bands. DNA was quantified by the use of a Kapa library quantification kit (Kapa Biosystems) for the final library before loading on a MiSeq (Illumina) flow cell (expected cluster density, 700,000 to 800,000/mm^2^) was performed with reagent kit v2 (Illumina) (500 cycles). We performed two runs of 251-bp paired-end sequencing, which yielded high-quality sequencing through the reading of each nucleotide of the V4 fragments twice after the assembly of read 1 and read 2. The raw sequence reads (.fastq format) have been deposited in the Dryad Digital Repository http://dx.doi.org/10.5061/dryad.m3p7d (41).

### Bioinformatic and taxonomic classification.

MiSeq datasets were processed with mothur v1.34 (42) and with the MiSeq standard operating procedure (SOP) (18). Briefly, the MiSeq SOP (http://www.mothur.org/wiki/MiSeq_SOP) allowed us to (i) construct contigs of paired-end read 1 and read 2 using the make.Contig command; (ii) remove the reads with poor quality of assembly (>275 bp); (iii) align unique sequences using Silva SSU Reference alignment v119 (89); (iv) remove the misaligned, nonspecific (eukaryotic), and chimeric reads (uchime program); (v) regroup the reads into operational taxonomic units (OTUs) with a 3% divergence threshold; and (vi) classify the OTUs using the Bayesian classifier included in mothur (bootstrap cutoff, 80%) and the Silva taxonomic file. At the end of the process, we obtained a table giving the number of reads for each OTU in each line and each PCR product in each column. For each OTU, the taxonomic classification (up to genus level) was provided. The abundance table generated by mothur for each PCR product and each OTU was filtered as described in Results and Discussion. The most abundant sequence for each OTU in each sample was extracted from the sequence data set with a custom-written Perl script (deposited in the Dryad Digital Repository [http://dx.doi.org/10.5061/dryad.m3p7d]) (41). The most abundant sequences for the 12 OTUs were deposited in GenBank (see below). The sequences were aligned with reference sequences from bacteria of the same genus available from Silva SSU Ref NR database v119 using SeaView v4 (91). We used a neighbor-joining method (bioNJ) to produce phylogenetic trees with a Kimura 2-parameter model using SeaView software, and species were identified on the basis of the “closest phylogenetic species.” We also used our sequences for blast analyses of GenBank (BLASTn against nucleotide collection [nr/nt] performed in January 2016) to identify the reference sequences to which they displayed the highest percentage of identity. The raw abundance table, the mothur command lines, the mothur output files, the Perl script, and the FASTA files used for the phylogenetic analyses have been deposited in the Dryad Digital Repository (http://dx.doi.org/10.5061/dryad.m3p7d) (41).

### Accession numbers.

The most abundant sequences for the 12 OTUs are available in GenBank (accession numbers KU697337 to KU697350).
