# Supplementary material for: 16S rRNA Amplicon Sequencing for Epidemiological Surveys of Bacteria in Wildlife
Source: mSystems. 2016 Jul 19;1(4):e00032-16. doi: 10.1128/mSystems.00032-16 (PMC5069956; doi:10.1128/mSystems.00032-16)
Supplement: Table S1 [file sys004162039st1.pdf]

**Table S1. Numbers of samples and numbers of PCRs for wild rodents and controls.** Negative Controls for dissection, $NC_{mus}$ ; Negative Controls for extraction, $NC_{ext}$ ; Negative Controls for PCR, $NC_{PCR}$ ; Negative Controls for indexing, $NC_{index}$ ; Positive Controls for PCR, $PC_{PCR}$ ; Positive Controls for Indexing, $PC_{alien}$. See also Figure 1 and the Materials & Methods section for more details concerning negative controls (NC) and positive controls (PC).

| MiSeq run | Types of samples | Number of samples | Number of PCRs* |
|---|---|---|---|
| Run 1 | Wild rodents | 355 | 790 |
| | $PC_{PCR}$: *Bartonella taylorii* (no dilution) | 1 | 2 |
| | $PC_{PCR}$/$PC_{alien}$: *Borrelia burgdorferi* (no dilution) | 1 | 2 |
| | $PC_{PCR}$/$PC_{alien}$: *Mycoplasma mycoides* (no dilution) | 1 | 4 |
| | $NC_{mus}$ | 4 | 8 |
| | $NC_{ext}$ | 4 | 8 |
| | $NC_{PCR}$ | / | 9 |
| Run 2 | Wild rodents | 356 | 712 |
| | $PC_{PCR}$: *Bartonella taylorii* (dilution: 1/100th) | 1 | 2 |
| | $PC_{PCR}$/$PC_{alien}$: *Borrelia burgdorferi* (dilution: 1/100th) | 1 | 2 |
| | $PC_{PCR}$/$PC_{alien}$: *Mycoplasma mycoides* (dilution: 1/100th) | 1 | 4 |
| | $NC_{ext}$ | 4 | 8 |
| | $NC_{PCR}$ | / | 9 |
| | $NC_{index}$ | / | 9 |
| | Total: | 729 | 1569 |

*PCR was performed in replicate for rodent samples and controls
