# Supplementary material for: 16S rRNA Amplicon Sequencing for Epidemiological Surveys of Bacteria in Wildlife
Source: mSystems. 2016 Jul 19;1(4):e00032-16. doi: 10.1128/mSystems.00032-16 (PMC5069956; doi:10.1128/mSystems.00032-16)
Supplement: Table S3 [file sys004162039st3.pdf]

Table S3. The 50 most abundant OTUs in wild rodents and controls. The twelve pathogenic OTUs from wild rodents are in bold and italic. The two OTUs from PC$_{alien}$ (PC$_{Borrelia_b}$ & PC$_{Mycoplasma_m}$) are highlighted in grey. A blank space was added at the end of the table to distinguish the first 50 most abundant OTUs and the *Mycoplasma*_OTU_6 and *Rickettsia*_OTU ranked in position 57 and 574 respectively.

							Run 1					Run 2				
OTU name	Phylum	Class	Order	Family	*Genus*	Total number of sequences (Run1 & Run 2)	Wild rodents	All Negative Controls (NC)	NC$_{ext}$ & NC$_{PCR}$	All Positive Controls (PC)	PC$_{alien}$ (PC$_{Borrelia_b}$ & PC$_{Mycoplasma_m}$)	Wild rodents	All Negative Controls (NC)	NC$_{ext}$ & NC$_{PCR}$	All Positive Controls (PC) (dilution 1/100th)	PC$_{alien}$ (PC$_{Borrelia_b}$ & PC$_{Mycoplasma_m}$) (dilution 1/100th)
Otu000001	Actinobacteria(100)	Actinobacteria(100)	Micrococcales(100)	Brevibacteriaceae(100)	*Brevibacterium(100)*	2 206 731	310	0	0	0	0	2 123 547	78 360	78 360	4 514	4 387
***Bartonella*_OTU**	**Proteobacteria(100)**	**Alphaproteobacteria(100)**	**Rhizobiales(100)**	**Bartonellaceae(100)**	***Bartonella(100)***	**1 761 155**	**67 973**	**3**	**2**	**134 151**	**27**	**1 547 652**	**3**	**3**	**11 373**	**76**
***Mycoplasma*_OTU_1**	**Tenericutes(100)**	**Mollicutes(100)**	**Mycoplasmatales(100)**	**Mycoplasmataceae(100)**	***Mycoplasma(100)***	**1 565 704**	**1 410 189**	**14**	**5**	**15**	**12**	**155 486**	**0**	**0**	**0**	**0**
***Mycoplasma*_OTU_2**	**Tenericutes(100)**	**Mollicutes(100)**	**Mycoplasmatales(100)**	**Mycoplasmataceae(100)**	***Mycoplasma(100)***	**1 036 084**	**0**	**0**	**0**	**0**	**0**	**1 035 890**	**193**	**193**	**1**	**1**
***Ehrlichia*_OTU**	**Proteobacteria(100)**	**Alphaproteobacteria(100)**	**Rickettsiales(100)**	**Anaplasmataceae(100)**	***Ehrlichia(100)***	**723 468**	**649 423**	**14**	**7**	**14**	**13**	**74 017**	**0**	**0**	**0**	**0**
Otu000004	Proteobacteria(100)	Gammaproteobacteria(100)	Pseudomonadales(100)	Pseudomonadaceae(100)	*Pseudomonas(100)*	700 806	645 274	50 018	41 700	469	465	4 864	178	178	3	3
***Mycoplasma*_OTU_3**	**Tenericutes(100)**	**Mollicutes(100)**	**Mycoplasmatales(100)**	**Mycoplasmataceae(100)**	***Mycoplasma(100)***	**634 967**	**507 369**	**2**	**2**	**5**	**3**	**127 590**	**1**	**1**	**0**	**0**
Otu000003	Actinobacteria(100)	Actinobacteria(100)	Micrococcales(100)	Dermabacteraceae(100)	*Brachybacterium(100)*	526 788	3 161	97	0	0	0	503 804	18 976	18 976	750	670
***Borrelia*_OTU**	**Spirochaetae(100)**	**Spirochaetes(100)**	**Spirochaetales(100)**	**Spirochaetaceae(100)**	***Borrelia(100)***	**367 509**	**345 845**	**20**	**11**	**8**	**7**	**21 636**	**0**	**0**	**0**	**0**
PC$_{Mycoplasma_m}$_OTU	Tenericutes(100)	Mollicutes(100)	Entomoplasmatales(100)	Entomoplasmataceae(100	*Incertae_Sedis(100)*	297 005	338	2	0	279 811	279 777	2	0	0	16 852	16 852
*Orientia*_OTU	Proteobacteria(100)	Alphaproteobacteria(100)	Rickettsiales(100)	Rickettsiaceae(100)	*Orientia(100)*	280 272	279 957	5	5	3	1	307	0	0	0	0
Otu000007	Actinobacteria(100)	Actinobacteria(100)	Corynebacteriales(100)	Dietziaceae(100)	*Dietzia(100)*	275 814	809	34	0	0	0	262 593	12 032	12 032	346	327
Otu000009	Proteobacteria(100)	Gammaproteobacteria(100)	Pseudomonadales(100)	Moraxellaceae(100)	*Acinetobacter(100)*	262 495	248 538	1 556	1 366	6	6	11 678	711	711	6	6
Otu000010	Firmicutes(100)	Bacilli(100)	Lactobacillales(100)	Lactobacillaceae(100)	*Lactobacillus(100)*	258 007	244 861	995	1	5	5	12 146	0	0	0	0
PC$_{Borrelia_b}$_OTU	Spirochaetae(100)	Spirochaetes(100)	Spirochaetales(100)	Spirochaetaceae(100)	*Borrelia(100)*	250 969	420	0	0	238 352	238 314	0	0	0	12 197	12 197
Otu000011	Firmicutes(100)	Bacilli(100)	Lactobacillales(100)	Lactobacillaceae(100)	*Lactobacillus(100)*	232 870	219 167	600	4	0	0	13 103	0	0	0	0
Otu000012	Firmicutes(100)	Bacilli(100)	Lactobacillales(100)	Lactobacillaceae(100)	*Lactobacillus(100)*	150 241	136 796	2 467	1	3	3	10 975	0	0	0	0
Otu000017	Proteobacteria(100)	Gammaproteobacteria(100)	Enterobacteriales(100)	Enterobacteriaceae(100)	*unclassified(70)*	91 877	43 644	114	114	827	827	47 261	30	30	1	1
***Mycoplasma*_OTU_4**	**Tenericutes(100)**	**Mollicutes(100)**	**Mycoplasmatales(100)**	**Mycoplasmataceae(100)**	***Mycoplasma(100)***	**85 596**	**0**	**0**	**0**	**0**	**0**	**85 583**	**13**	**13**	**0**	**0**
Otu000018	Proteobacteria(100)	Gammaproteobacteria(100)	Pasteurellales(100)	Pasteurellaceae(100)	*unclassified(83)*	84 403	78 632	1 894	1 770	3	2	3 872	0	0	2	2
Otu000024	Firmicutes(100)	Bacilli(100)	Lactobacillales(100)	Leuconostocaceae(100)	*Weissella(100)*	82 171	77 033	50	4	3	3	5 085	0	0	0	0
Otu000028	Proteobacteria(100)	Betaproteobacteria(100)	Burkholderiales(100)	Oxalobacteraceae(100)	*Herbaspirillum(69)*	74 189	65 973	5 435	5 284	37	37	2 696	46	46	2	2
Otu000016	Proteobacteria(100)	Betaproteobacteria(100)	Burkholderiales(100)	Comamonadaceae(100)	*Pelomonas(75)*	73 751	50 487	8 966	7 973	64	64	12 874	1 350	1 350	10	10
Otu000023	Proteobacteria(100)	Epsilonproteobacteria(100)	Campylobacterales(100)	Helicobacteraceae(100)	*Helicobacter(97)*	72 142	71 317	0	0	1	1	824	0	0	0	0
Otu000019	Proteobacteria(100)	Gammaproteobacteria(100)	Enterobacteriales(100)	Enterobacteriaceae(100)	*unclassified(98)*	69 999	69 010	4	1	0	0	985	0	0	0	0
Otu000021	Proteobacteria(100)	Gammaproteobacteria(100)	Enterobacteriales(100)	Enterobacteriaceae(100)	*Yersinia(95)*	62 633	57 413	4 658	4 416	74	74	488	0	0	0	0
Otu000032	Bacteroidetes(100)	Bacteroidia(100)	Bacteroidales(100)	Bacteroidaceae(100)	*Bacteroides(100)*	56 644	11 247	1	1	0	0	45 396	0	0	0	0
***Mycoplasma*_OTU_5**	**Tenericutes(100)**	**Mollicutes(100)**	**Mycoplasmatales(100)**	**Mycoplasmataceae(100)**	***Mycoplasma(100)***	**56 324**	**0**	**0**	**0**	**0**	**0**	**56 324**	**0**	**0**	**0**	**0**
Otu000030	Bacteroidetes(100)	Bacteroidia(100)	Bacteroidales(100)	Bacteroidaceae(100)	*Bacteroides(100)*	50 179	46 668	18	0	1	0	3 492	0	0	0	0
Otu000025	Firmicutes(100)	Bacilli(100)	Lactobacillales(100)	Lactobacillaceae(100)	*Lactobacillus(100)*	46 332	41 591	266	1	0	0	4 475	0	0	0	0
Otu000027	Firmicutes(100)	Bacilli(100)	Bacillales(100)	Bacillaceae(100)	*Geobacillus(97)*	42 581	41 128	1 179	5	0	0	271	0	0	3	3
Otu000036	Firmicutes(100)	Bacilli(100)	Lactobacillales(100)	Streptococcaceae(100)	*Streptococcus(100)*	39 271	31 561	6 137	5 309	0	0	1 568	2	2	3	2
Otu000026	Proteobacteria(100)	Gammaproteobacteria(100)	Pseudomonadales(100)	Moraxellaceae(100)	*Acinetobacter(100)*	38 173	33 294	797	580	15	15	3 443	67	67	557	557
Otu000022	Firmicutes(100)	Bacilli(100)	Bacillales(100)	Staphylococcaceae(100)	*Staphylococcus(67)*	35 526	22 065	923	515	0	0	11 996	527	527	15	10
Otu000038	Actinobacteria(100)	Actinobacteria(100)	Bifidobacteriales(100)	Bifidobacteriaceae(100)	*Bifidobacterium(100)*	35 027	31 814	212	1	0	0	3 001	0	0	0	0
Otu000142	Proteobacteria(100)	Gammaproteobacteria(100)	Aeromonadales(100)	Aeromonadaceae(100)	*Aeromonas(100)*	34 429	34 331	0	0	0	0	98	0	0	0	0
Otu000061	Proteobacteria(100)	Deltaproteobacteria(100)	Myxococcales(100)	Myxococcaceae(96)	*unclassified(96)*	32 821	32 669	42	0	1	0	109	0	0	0	0
***Streptobacillus*_OTU**	**Fusobacteria(100)**	**Fusobacteriia(100)**	**Fusobacteriales(100)**	**Leptotrichiaceae(100)**	***Streptobacillus(100)***	**32 399**	**0**	**0**	**0**	**0**	**0**	**32 399**	**0**	**0**	**0**	**0**
Otu000039	Proteobacteria(100)	Alphaproteobacteria(100)	Caulobacterales(100)	Caulobacteraceae(100)	*Brevundimonas(100)*	23 719	15 160	3 778	3 672	0	0	4 306	473	473	2	2
Otu000125	Bacteroidetes(100)	Bacteroidia(100)	Bacteroidales(100)	Prevotellaceae(100)	*Alloprevotella(100)*	23 197	22 943	43	0	0	0	211	0	0	0	0
Otu000049	Deinococcus-Thermus(100	Deinococci(100)	Thermales(100)	Thermaceae(100)	*Meiothermus(100)*	23 074	21 780	964	1	0	0	330	0	0	0	0
Otu000051	Proteobacteria(100)	Epsilonproteobacteria(100)	Campylobacterales(100)	Helicobacteraceae(100)	*Helicobacter(99)*	22 844	4 985	0	0	0	0	17 859	0	0	0	0
Otu000070	Bacteroidetes(100)	Bacteroidia(100)	Bacteroidales(100)	Prevotellaceae(100)	*Prevotella(99)*	22 649	19 711	890	2	0	0	2 048	0	0	0	0
Otu000045	Proteobacteria(100)	Gammaproteobacteria(100)	Enterobacteriales(100)	Enterobacteriaceae(100)	*Proteus(99)*	22 344	87	0	0	0	0	22 257	0	0	0	0
Otu000033	Proteobacteria(100)	Alphaproteobacteria(100)	Sphingomonadales(100)	Sphingomonadaceae(60)	*Sphingomonas(59)*	19 819	15 822	519	246	13	13	3 330	135	135	0	0
Otu000058	Proteobacteria(100)	Gammaproteobacteria(100)	Xanthomonadales(100)	Xanthomonadaceae(100)	*Luteimonas(99)*	16 794	15 899	722	2	0	0	173	0	0	0	0
Otu000076	Bacteroidetes(100)	Bacteroidia(100)	Bacteroidales(100)	S24-7(100)	*unclassified(100)*	16 544	15 459	206	1	0	0	879	0	0	0	0
Otu000040	Firmicutes(100)	Bacilli(100)	Bacillales(100)	Planococcaceae(98)	*unclassified(58)*	16 005	12 278	1 819	1	0	0	1 901	7	7	0	0
Otu000050	Bacteroidetes(100)	Bacteroidia(100)	Bacteroidales(100)	Bacteroidaceae(100)	*Bacteroides(100)*	15 883	14 752	0	0	0	0	1 131	0	0	0	0
Otu000043	Firmicutes(100)	Clostridia(100)	Clostridiales(100)	Ruminococcaceae(100)	*unclassified(99)*	15 284	14 210	127	0	0	0	947	0	0	0	0
Mycoplasma*_OTU_6	**Tenericutes(100)**	**Mollicutes(100)**	**Mycoplasmatales(100)**	**Mycoplasmataceae(100)**	***Mycoplasma(100)***	**13 356**	**0**	**0**	**0**	**0**	**0**	**13 356**	**0**	**0**	**0**	**0**
Rickettsia*_OTU*	**Proteobacteria(100)**	**Alphaproteobacteria(100)**	**Rickettsiales(100)**	**Rickettsiaceae(100)**	***Rickettsia(100)***	**593**	**4**	**0**	**0**	**0**	**0**	**589**	**0**	**0**	**0**	**0**
Other OTUs	/	/	/	/	/	1 668 136	1 376 047	57 967	12 205	3 650	438	228 260	1 461	1 461	751	138
					Total number of sequences:	14 647 593	7 149 444	153 558	85 208	657 531	520 107	6 525 107	114 565	114 565	47 388	35 246

* *Mycoplasma*_OTU_6 is ranked in position 57
** *Rickettsia*_OTU is ranked in position 574
