# Supplementary material for: 16S rRNA Amplicon Sequencing for Epidemiological Surveys of Bacteria in Wildlife
Source: mSystems. 2016 Jul 19;1(4):e00032-16. doi: 10.1128/mSystems.00032-16 (PMC5069956; doi:10.1128/mSystems.00032-16)

Figure S1. Taxonomic assignment of the V4 16S rRNA sequences in wild rodents and in negative controls for extraction and of PCR. The histograms show the percentage of sequences for the most abundant bacterial genera in the MiSeq run 1 and run 2. Notice the presence of several bacterial genera in the controls, which were likely due to the inherent contamination of laboratory reagents by bacterial DNA and which are thereafter called contaminant genera. These contaminant genera are also present (in lower percentage) in the rodent samples. The different in bacterial contaminant composition between run 1 and run 2 reflects the use of different kits manufactured at several months apart (Qiagen technical service, pers. com.). The differences in the pathogenic bacteria proportions and compositions between run 1 and run 2 reflects the different origins of the samples (A) run 1: *Mastomys erythroleucus* (n=148) and *Mus musculus* (n=207) from the north Senegal ; (B) run 2: *Mastomys erythroleucus* (n=73), *Mastomys natalensis* (n=93) et *Rattus rattus* (n=190) from the south Senegal).

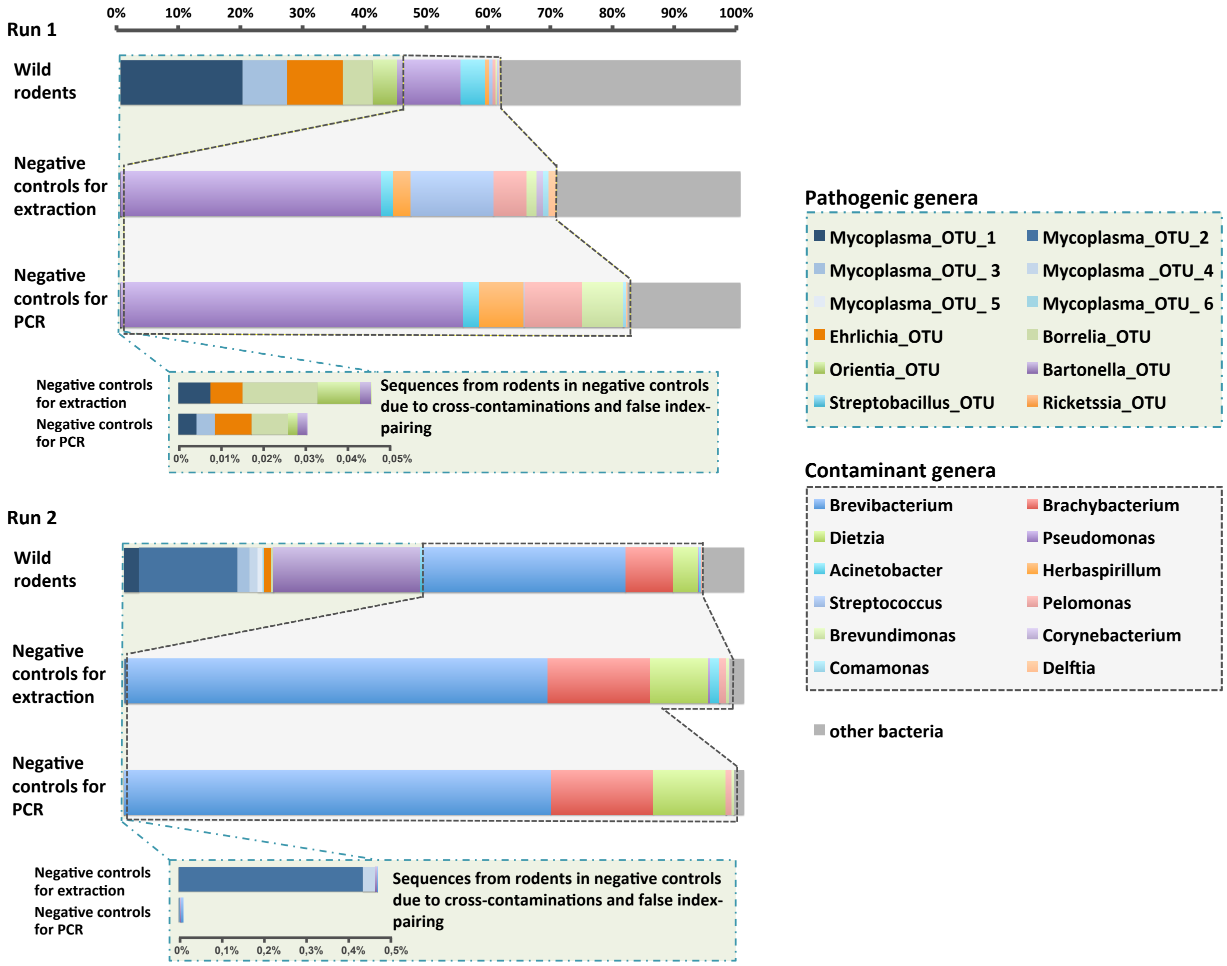

Supplement: Figure S1 [file sys004162039sf7.pdf]
