# Supplementary material for: 16S rRNA Amplicon Sequencing for Epidemiological Surveys of Bacteria in Wildlife
Source: mSystems. 2016 Jul 19;1(4):e00032-16. doi: 10.1128/mSystems.00032-16 (PMC5069956; doi:10.1128/mSystems.00032-16)
Supplement: Table S4 [file sys004162039st4.pdf]

Table S4. Bacterial contaminants observed in negative ad positive controls. They were identified as contaminants on the basis of negative controls for extraction and PCR. Taxa in bold correspond to the sequences of DNA extracted from laboratory isolates.

Run name	Negative and positive controls (no. of PCR replicates)	Number of sequences				Taxon (frequency)
		Total	Mean	Min.	Max.	
Run 1	*Bartonella taylorii* (*n*=2), no dilution	137424	68712	64290	73134	***Bartonella* (0.975)**, *Propionibacterium* (0.023), other bacteria (0.002)
	Borrelia burgdorferi (*n*=2), no dilution	239465	119733	118913	120552	***Borrelia* (0.995)**, other bacteria (0.005)
	Mycoplasma mycoides (*n*=4), no dilution	280642	70161	58896	82933	**Entomoplasmataceae° (0.997)**, other bacteria (0.003)
	NC_{ext} (*n*=8)	39308	4914	2843	8967	*Pseudomonas** (0.42), *Streptococcus** (0.134), *Pelomonas** (0.054), *Haemophilus* (0.042), *Yersinia* (0.029), *Herbaspirillum** (0.028), *Granulicatella* (0.02), *Acinetobacter** (0.019), *Actinomyces* (0.017), *Brevundimonas** (0.016), *Veillonella* (0.013), *Staphylococcus* (0.013), *Delftia** (0.013), Comamonadaceae* (0.012), Pasteurellaceae (0.012), *Porphyromonas* (0.011), *Corynebacterium** (0.011), *Gemella* (0.01), *other bacteria (0.126)*
	NC_{mus} (*n*=8)	68350	8544	32*	26211	*Pseudomonas** (0.121), *Lactobacillus* (0.063), Bacillales* (0.037), Planococcaceae (0.033), *Microvirga* (0.031), Bacteroidales (0.028), Thermomicrobia (0.027), Lachnospiraceae (0.027), Nonomuraea (0.026), *Geodermatophilus** (0.023), *Sphingobacterium* (0.022), *Prevotella* (0.022), *Blautia* (0.019), *Pseudonocardia* (0.017), Geodermatophilaceae* (0.017), *Geobacillus* (0.017), *Meiothermus* (0.014), *Defluviimonas* (0.013), *Streptococcus** (0.013), *Pelomonas** (0.012), *Luteimonas* (0.01), other bacteria (0.408)
	NC_{PCR} (*n*=9)	45900	5100	3144	8002	*Pseudomonas** (0.552), *Pelomonas** (0.092), *Herbaspirillum** (0.072), *Brevundimonas** (0.067), *Yersinia* (0.065), *Acinetobacter** (0.026), other bacteria (0.125)
Run 2	*Bartonella taylorii* (*n*=2), dilution: 1/100th	12142	6071	4624	7518	***Bartonella* (0.928)**, *Propionibacterium* (0.042), *Brevibacterium**^ (0.013), other bacteria (0.017)
	Borrelia burgdorferi (*n*=2), dilution: 1/100th	13378	6689	6214	7164	***Borrelia* (0.912)**, *Acinetobacter** (0.046), *Brevibacterium**^ (0.036), other bacteria (0.006)
	Mycoplasma mycoides (*n*=4), dilution: 1/100th	21868	5467	4104	6520	**Entomoplasmataceae° (0.771)**, *Brevibacterium**^ (0.179), *Brachybacterium*^ (0.028), *Dietzia**^ (0.014), other bacteria (0.007)
	NC_{ext} (*n*=8)	53334	6667	5275	7669	*Brevibacterium**^ (0.679), *Brachybacterium*^ (0.166), *Dietzia**^ (0.093), *Acinetobacter** (0.015), *Pelomonas** (0.011), other bacteria (0.036)
	NC_{index} (*n*=9)	52	6	1	12	NA
	NC_{PCR} (*n*=8)	61231	7654	5855	9145	*Brevibacterium**^ (0.689), *Brachybacterium*^ (0.165), *Dietzia**^ (0.117), other bacteria (0.029)

° sequences of *Mycoplasma mycoides* were identified as Entomoplasmataceae due to a frequent taxonomic error present in most databases [44]
* taxa identified as reagent contaminants by Salter *et al*. [23]
^ taxa identified as PCR kit contaminants (Qiagen, personal communication)
