# Supplementary material for: 16S rRNA Amplicon Sequencing for Epidemiological Surveys of Bacteria in Wildlife
Source: mSystems. 2016 Jul 19;1(4):e00032-16. doi: 10.1128/mSystems.00032-16 (PMC5069956; doi:10.1128/mSystems.00032-16)
Supplement: Table S5 [file sys004162039st5.pdf]

Table S5. Proportion of sequences and proportion of positive results removed at each step in data filtering. Note that several positive results may be recorded for the same rodent in cases of co-infection.

	OTUs of interest	Sequences*					Positive results				
		No. before filtering	% removed from previous step			% removed (total)	No. before filtering	% removed from previous step			% removed (total)
			T_{CC}	T_{FA}	PCR Replicates			T_{CC}	T_{FA}	PCR Replicates	
Run 1	Mycoplasma_OTU_1	1226193	0,01%	0,36%	0,14%	0,51%	297	22%	78%	4%	83%
	Mycoplasma_OTU_3	507237	0,02%	0,27%	0,06%	0,35%	265	20%	75%	4%	80%
	Ehrlichia_OTU	644244	0,04%	0,34%	0,17%	0,55%	283	36%	72%	8%	83%
	Borrelia_OTU	319305	0,14%	0,34%	0,03%	0,50%	238	69%	62%	4%	89%
	Orientia_OTU	242299	0,04%	0,25%	0,40%	0,69%	199	36%	59%	12%	77%
	Bartonella_OTU	67921	0,07%	0,71%	0,14%	0,91%	124	32%	87%	18%	93%
Run 2	Mycoplasma_OTU_1	155486	0,00%	0,10%	0,00%	0,10%	74	0%	31%	0%	31%
	Mycoplasma_OTU_2	1035890	0,10%	0,05%	0,03%	0,18%	177	47%	3%	1%	49%
	Mycoplasma_OTU_3	127590	0,00%	0,13%	0,26%	0,40%	103	6%	10%	5%	19%
	Mycoplasma_OTU_4	85583	0,08%	0,04%	0,29%	0,41%	30	27%	0%	14%	37%
	Mycoplasma_OTU_5	56324	0,00%	0,12%	0,17%	0,29%	26	0%	38%	31%	58%
	Mycoplasma_OTU_6	13356	0,00%	0,01%	0,00%	0,01%	17	0%	6%	0%	6%
	Ehrlichia_OTU	74017	0,00%	0,05%	0,05%	0,09%	24	0%	38%	13%	46%
	Borrelia_OTU	21636	0,00%	0,05%	0,09%	0,13%	15	0%	33%	20%	47%
	Orientia_OTU	307	0,00%	0,00%	7,17%	7,17%	5	0%	0%	60%	60%
	Bartonella_OTU	1547652	0,01%	0,22%	0,19%	0,42%	246	26%	24%	4%	47%
	Streptobacillus_OTU	32399	0,00%	0,06%	0,46%	0,52%	29	0%	17%	33%	45%
	Rickettsia_OTU	589	0,00%	0,00%	0,34%	0,34%	3	0%	0%	33%	33%

*:sum of sequences in both duplicates

T_{CC} based on the maximum number of sequences observed in a control for each OTU in each run

T_{FA} based on the false assignment rate (0.02%) weighted by the total number of sequences for each OTU in each run
