# Supplementary material for: 16S rRNA Amplicon Sequencing for Epidemiological Surveys of Bacteria in Wildlife
Source: mSystems. 2016 Jul 19;1(4):e00032-16. doi: 10.1128/mSystems.00032-16 (PMC5069956; doi:10.1128/mSystems.00032-16)
Supplement: Figure S2 [file sys004162039sf8.pdf]

Figure S2. Numbers of sequences of the positive controls for indexing PC$_{Borrelia_b}$ (in blue) and PC$_{Mycoplasma_m}$ (in red) in the various PCR products, with a dual-indexing design, for MiSeq runs 1 (a) and 2 (b). The two PCRs for PC$_{Borrelia_b}$ were performed with 96-well microplate 9, positions A1 and E1 for run 1 and B1 and F1 for run 2, and the four PCRs for PC$_{Mycoplasma_m}$ were performed with 96-well microplate 9, positions C1, D1, G1 and H1 for the two runs. The numbers of sequences for the other wells correspond to indexing mistakes due to false index-pairing due to mixed clusters during the sequencing (see Table 1).

Index i7 name	Index i7 sequence	Reverse complement i7 sequence
SA701	AACTCTCG	CGAGAGTT
SA702	ACTATGTC	GACATAGT
SA703	AGTAGCGT	ACGCTACT
SA704	CAGTGAGT	ACTCACTG
SA705	CGTACTCA	TGAGTACG
SA706	CTACGCAG	CTGCGTAG
SA707	GGAGACTA	TAGTCTCC
SA708	GTCGCTCG	CGAGCGAC
SA709	GTCGTAGT	ACTACGAC
SA710	TAGCAGAC	GTCTGCTA
SA711	TCATAGAC	GTCTATGA
SA712	TCGCTATA	TATAGCGA
SB701	AAGTCGAG	CTCGACTT
SB702	ATACTTCG	CGAAGTAT
SB703	AGCTGCTA	TAGCAGCT
SB704	CATAGAGA	TCTCTATG
SB705	CGTAGATC	GATCTACG
SB706	CTCGTTAC	GTAACGAG
SB707	GCGCACGT	ACGTGCGC
SB708	GGTACTAT	ATAGTACC
SB709	GTATACGC	GCGTATAC
SB710	TACGAGCA	TGCTCGTA
SB711	TCAGCGTT	AACGCTGA
SB712	TCGCTACG	CGTAGCGA
SC701	ACCTACTG	CAGTAGGT
SC702	AGCGCTAT	ATAGCGCT
SC703	AGTCTAGA	TCTAGACT
SC704	CATGAGGA	TCCTCATG
SC705	CTAGCTCG	CGAGCTAG
SC706	CTCTAGAG	CTCTAGAG
SC707	GAGCTCAT	ATGAGCTC
SC708	GGTATGCT	AGCATACC
SC709	GTATGACG	CGTCATAC
SC710	TAGACTGA	TCAGTCTA
SC711	TCACGATG	CATCGTGA
SC712	TCGAGCTC	GAGCTCGA

Index i5 name	Index i5 sequence	Row
SA501	ATCGTACG	A
SA502	ACTATCTG	B
SA503	TAGCGAGT	C
SA504	CTGCGTGT	D
SA505	TCATCGAG	E
SA506	CGTGAGTG	F
SA507	GGATATCT	G
SA508	GACACCGT	H
SB501	CTACTATA	A
SB502	CGTTACTA	B
SB503	AGAGTCAC	C
SB504	TACGAGAC	D
SB505	ACGTCTCG	E
SB506	TCGACGAG	F
SB507	GATCGTGT	G
SB508	GTCAGATA	H
SC501	ACGACGTG	A
SC502	ATATACAC	B
SC503	CGTCGCTA	C
SC504	CTAGAGCT	D
SC505	GCTCTAGT	E
SC506	GACACTGA	F
SC507	TGCGTACG	G
SC508	TAGTGTAG	H

(a) Run 1

Plate 1

	1	2	3	4	5	6	7	8	9	10	11	12
A												
B												
C												
D												
E												
F												
G												
H												

Plate 2

	1	2	3	4	5	6	7	8	9	10	11	12
A												
B												
C												
D												
E												
F												
G												
H												

Plate 3

	1	1	2	3	4	5	6	7	8	9	10	11	12
A	18	21											
B	13	29											
C	17	31											
D	3	24											
E	15	11											
F	12	10											
G	11	17											
H	18	8											

Plate 4

	1	2	3	4	5	6	7	8	9	10	11	12
A												
B												
C												
D												
E												
F												
G												
H												

Plate 5

	1	2	3	4	5	6	7	8	9	10	11	12
A												
B												
C												
D												
E												
F												
G												
H												

Plate 6

	1	1	2	3	4	5	6	7	8	9	10	11	12
A	17	34											
B	23	43*											
C	20	34											
D	28*	24											
E	4	16											
F	5	18											
G	6	16											
H	24	25											

Plate 7

	1	2	3	4	5	6	7	8	9	10	11	12
A	1	0	0	0	0	0	0	0	0	0	0	1
B												
C	0	2	1	0	3	1	0	0	0	2	2	1
D	0	1	2	0	3	0	0	0	0	0	2	0
E	1	1	3	1	5	2	0	0	0	0	0	0
F												
G	0	1	0	1	4	3	0	0	3	0	0	0
H	0	0	0	2	3	3	1	0	0	0	0	0

Plate 8

	1	2	3	4	5	6	7	8	9	10	11	12
A	0	0	0	0	3	3	0	1	0	1	0	0
B												
C	0	2	0	3	4	0	0	0	0	0	0	0
D	0	1	1	4	0	0	0	0	0	0	0	0
E	1	0	1	1	5	2	0	3	0	0	1	0
F												
G	0	0	4	0	1	1	1	1	0	1	0	0
H	2	0	6	0	2	0	0	0	0	0	3	0

Plate 9

	1	1	2	3	4	5	6	7	8	9	10	11	12
A	18	**118652**	1	2	1	3	1	1	1	0	0	4	0
B	14	17											
C	**82767**	21	0	0	0	0	1	0	0	0	0	3	2
D	**73320**	21	0	0	0	0	0	2	1	3	0	0	0
E	6	**119586**	1	0	2	0	1	2	2	0	0	0	0
F	20	21											
G	**64959**	11	0	0	0	0	1	1	0	0	1	0	0
H	**58707**	23	1	0	0	1	1	2	0	2	1	0	0

(b) Run 2

(Index i7 and i5 names and sequences as above.)

Plate 1

	1	2	3	4	5	6	7	8	9	10	11	12
A												
B												
C												
D												
E												
F												
G												
H												

Plate 2

	1	2	3	4	5	6	7	8	9	10	11	12
A												
B												
C												
D												
E												
F												
G												
H												

Plate 3

	1	1	2	3	4	5	6	7	8	9	10	11	12
A													
B													
C													
D													
E													
F													
G													
H													

Plate 4

	1	2	3	4	5	6	7	8	9	10	11	12
A												
B												
C												
D												
E												
F												
G												
H												

Plate 5

	1	2	3	4	5	6	7	8	9	10	11	12
A												
B												
C												
D												
E												
F												
G												
H												

Plate 6

	1	1	2	3	4	5	6	7	8	9	10	11	12
A													
B													
C													
D													
E													
F													
G													
H													

Plate 7

	1	2	3	4	5	6	7	8	9	10	11	12
A												
B												
C												
D												
E												
F												
G												
H												

Plate 8

	1	2	3	4	5	6	7	8	9	10	11	12
A												
B												
C												
D												
E												
F												
G												
H												

Plate 9

	1	1	2	3	4	5	6	7	8	9	10	11	12
A													
B		**6426**	1							1			
C	**5766**												1
D	**2568**												
E													
F		**5771**											
G	**5055**					1							
H	**3463**												
