# Supplementary material for: 16S rRNA Amplicon Sequencing for Epidemiological Surveys of Bacteria in Wildlife
Source: mSystems. 2016 Jul 19;1(4):e00032-16. doi: 10.1128/mSystems.00032-16 (PMC5069956; doi:10.1128/mSystems.00032-16)

Figure S3. Plots of the number of sequences (log (x+1) scale) from bacterial OTUs in both PCR replicates (PCR1 & PCR2) for the 356 wild rodents analyzed in the second MiSeq run. Note that each rodent was tested with two replicate PCRs. Green points correspond to rodents with two positive results after the filtering process; red points correspond to rodents with one positive result and one negative result; and blue points correspond to rodents with two negative results. The light blue area and lines correspond to the threshold values used for the data filtering: samples below the lines are filtered out. See Figure 4 for plots corresponding to the first MiSeq run.

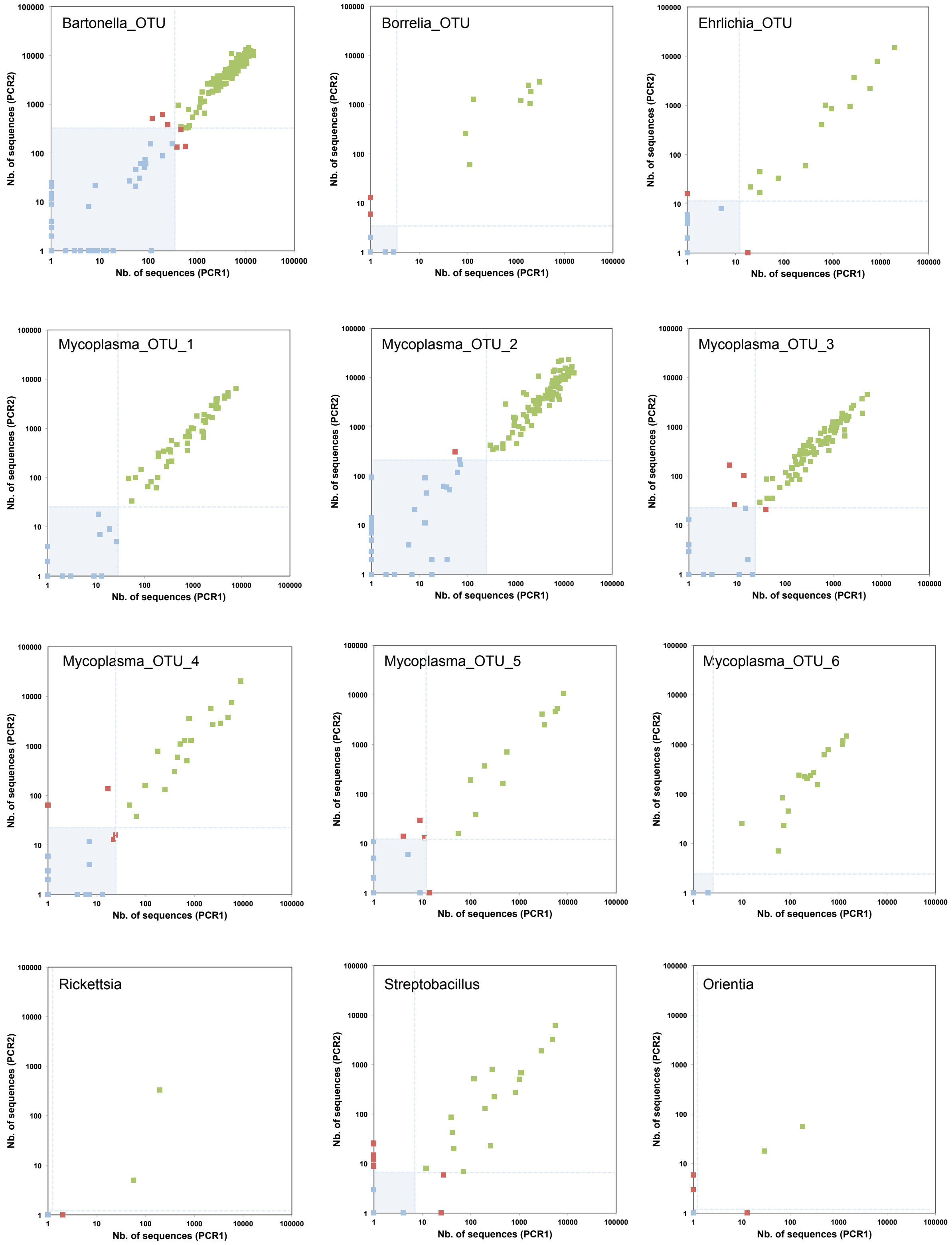

Supplement: Figure S3 [file sys004162039sf9.pdf]
