# Supplementary material for: 16S rRNA Amplicon Sequencing for Epidemiological Surveys of Bacteria in Wildlife
Source: mSystems. 2016 Jul 19;1(4):e00032-16. doi: 10.1128/mSystems.00032-16 (PMC5069956; doi:10.1128/mSystems.00032-16)
Supplement: Table S6 [file sys004162039st6.pdf]

Table S6. Proportion of positive results for both PCR products at each step in data filtering.
Note that several positive results may be recorded for the same rodent in cases of co-infection.

	OTUs of interest	% of rodents positive for both PCR replicates		
		Before filtering	T_{CC}	T_{FA}
**Run 1**	Mycoplasma_OTU_1	68%	64%	96%
	Mycoplasma_OTU_3	49%	46%	96%
	Ehrlichia_OTU	56%	56%	92%
	Borrelia_OTU	38%	53%	96%
	Orientia_OTU	43%	54%	88%
	Bartonella_OTU	19%	20%	82%
**Run 2**	Mycoplasma_OTU_1	76%	76%	100%
	Mycoplasma_OTU_2	59%	96%	99%
	Mycoplasma_OTU_3	86%	92%	95%
	Mycoplasma_OTU_4	77%	91%	82%
	Mycoplasma_OTU_5	62%	62%	69%
	Mycoplasma_OTU_6	94%	94%	100%
	Ehrlichia_OTU	58%	58%	87%
	Borrelia_OTU	53%	53%	80%
	Orientia_OTU	40%	40%	40%
	Bartonella_OTU	66%	83%	96%
	Streptobacillus_OTU	59%	59%	67%
	Rickettsia_OTU	67%	67%	67%

T_{CC} based on the maximum number of sequences observed in a control for each OTU in each run
T_{FA} based on the false assignment rate (0.02%) weighted by the total number of sequences for each OTU in each run
