# Supplementary material for: 16S rRNA Amplicon Sequencing for Epidemiological Surveys of Bacteria in Wildlife
Source: mSystems. 2016 Jul 19;1(4):e00032-16. doi: 10.1128/mSystems.00032-16 (PMC5069956; doi:10.1128/mSystems.00032-16)
Supplement: Figure S4 [file sys004162039sf10.pdf]

Figure S4. Phylogenetic trees of the 16S rRNA V4 sequences for 12 pathogenic bacterial OTUs detected in wild rodents from Senegal. Sequences boxed with an orange line were retrieved from African rodents and/or corresponds to positive controls (PC) for *Borellia burgdorferi*, *Mycoplasma mycoides* and *Bartonella taylorii*. The other sequences were extracted from the SILVA database and GenBank. Trees include all lineages collected for *Rickettsia*, *Bartonella*, *Ehrlichia* and *Orientia*, but only lineages of the Spotted Fever Group for *Borrelia*, and lineages of the pneumonia group for *Mycoplasma*. The numbers indicated are the bootstrap values >55%. Fasta files used have been deposited in the Dryad Digital Repository: http://dx.doi.org/10.5061/dryad.m3p7d.

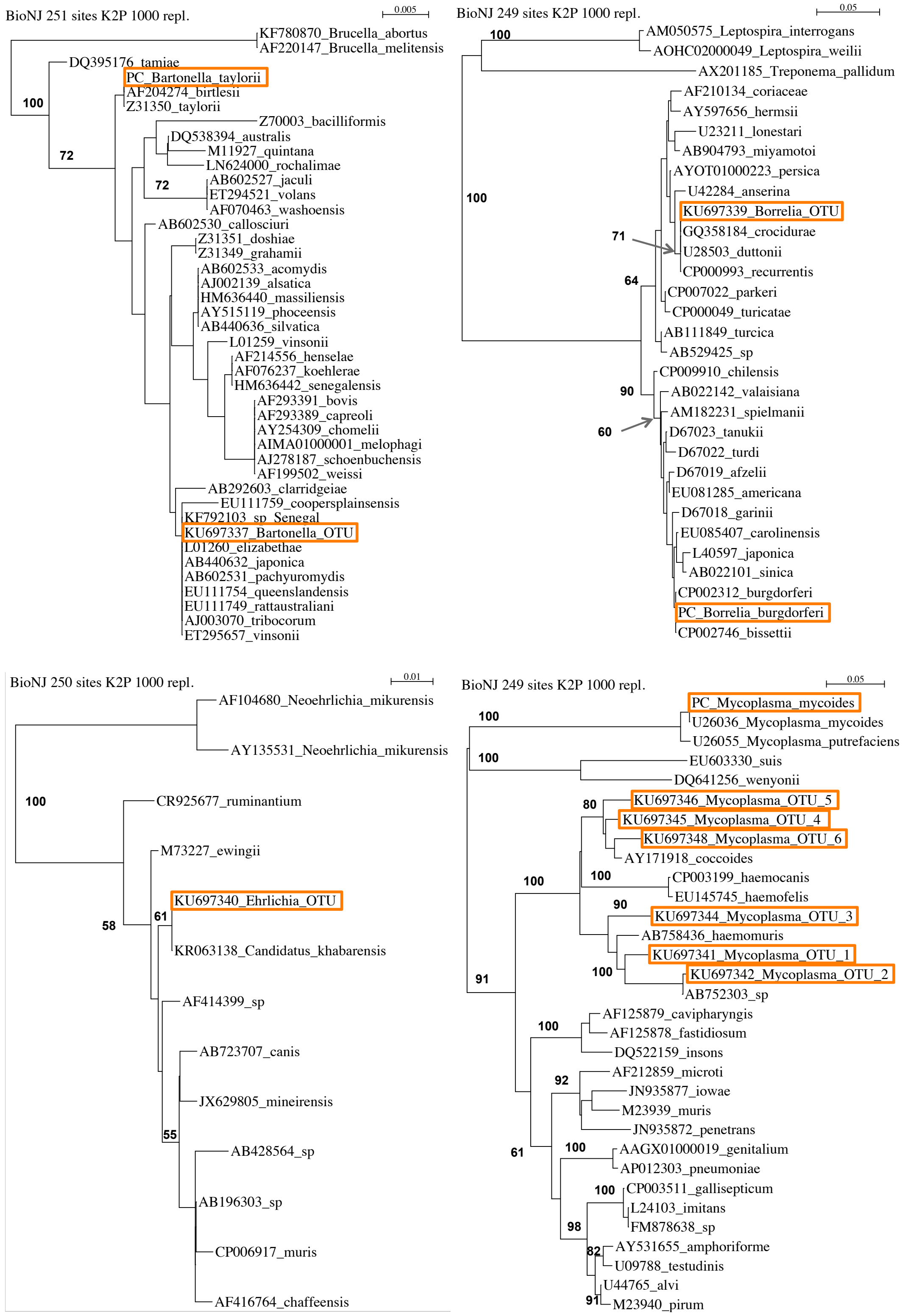

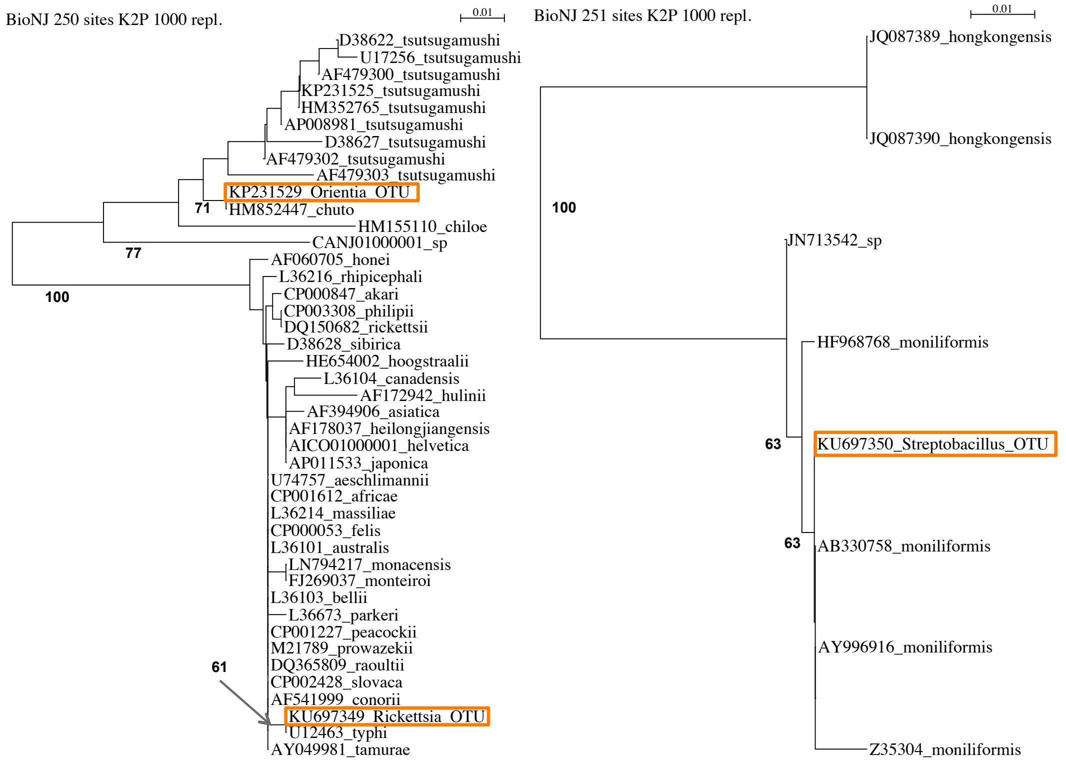
BioNJ 250 sites K2P 1000 repl.
0.01
D38622_tsutsugamushi
U17256_tsutsugamushi
AF479300_tsutsugamushi
KP231525_tsutsugamushi
HM352765_tsutsugamushi
AP008981_tsutsugamushi
D38627_tsutsugamushi
AF479302_tsutsugamushi
AF479303_tsutsugamushi
KP231529_Orientia_OTU
71
HM852447_chuto
HM155110_chiloe
CANJ01000001_sp
77
100
AF060705_honei
L36216_rhipicephali
CP000847_akari
CP003308_philipii
DQ150682_rickettsii
D38628_sibirica
HE654002_hoogstraalii
L36104_canadensis
AF172942_hulinii
AF394906_asiatica
AF178037_heilongjiangensis
AICO01000001_helvetica
AP011533_japonica
U74757_aeschlimannii
CP001612_africae
L36214_massiliae
CP000053_felis
L36101_australis
LN794217_monacensis
FJ269037_monteiroi
L36103_bellii
L36673_parkeri
CP001227_peacockii
M21789_prowazekii
DQ365809_raoultii
61
CP002428_slovaca
AF541999_conorii
KU697349_Rickettsia_OTU
U12463_typhi
AY049981_tamurae
BioNJ 251 sites K2P 1000 repl.
0.01
JQ087389_hongkongensis
JQ087390_hongkongensis
100
JN713542_sp
HF968768_moniliformis
63
KU697350_Streptobacillus_OTU
63
AB330758_moniliformis
AY996916_moniliformis
Z35304_moniliformis
